# Experimental and Computational Studies on Bio-Inspired Flavylium Salts as Sensitizers for Dye-Sensitized Solar Cells

**DOI:** 10.3390/ma15196985

**Published:** 2022-10-08

**Authors:** Iulia Păușescu, Anamaria Todea, Diana-Maria Dreavă, Tania Boboescu, Bianca Pațcan, Larisa Pațcan, Daiana Albulescu, Valentin Badea, Francisc Peter, Róbert Tőtős, Daniel Ursu, Lorant Szolga, Mihai Medeleanu

**Affiliations:** 1 Faculty of Industrial Chemistry and Environmental Engineering, Politehnica University of Timişoara, Carol Telbisz 6, 300001 Timisoara, Romania; 2 National Institute of Research and Development for Electrochemistry and Condensed Matter, Dr A. Păunescu Podeanu 144, 300569 Timisoara, Romania; 3 Faculty of Chemistry and Chemical Engineering, Babes Bolyai University, Arany Janos 11, 400028 Cluj-Napoca, Romania; 4 Optoelectronics Group, Base of Electronics Department, ETTI, Technical University of Cluj-Napoca, 28 Memorandumului Str., 400114 Cluj-Napoca, Romania

**Keywords:** flavylium dyes, photochromism, density functional theory, DSSC, photovoltaic parameters

## Abstract

Six new bio-inspired flavylium salts were synthesized and investigated by a combined computational and experimental study for dye-sensitized solar cell applications. The compounds were characterized by FT–IR, UV–Vis, NMR spectroscopy, and LC–MS spectrometry techniques. The pH-dependent photochromic properties of the flavylium dyes were investigated through a UV–Vis spectroscopy study and revealed that they follow the same network of chemical reactions as anthocyanins upon pH changes. The structural and electronic properties of the dyes were investigated using density functional theory (DFT) and time-dependent density functional theory (TD–DFT). Geometry optimization calculation revealed that all dyes, regardless of the specie, flavylium cations or quinoidal bases, present a planar geometry. The photovoltaic performances of the dyes, in both flavylium and quinoidal base forms, were evaluated by the HOMO and LUMO energies and by calculating the light-harvesting efficiencies, the free energy change of electron injection, and the free energy change regeneration. The MO analysis showed that all dyes can inject electrons into the conduction band of the TiO_2_ upon excitation and that the redox couple can regenerate the oxidized dyes. The results obtained for the free energy change of electron injection suggest that the quinoidal bases should inject electrons into the semiconductor more efficiently than the flavylium cations. The values for the free energy change regeneration showed that the redox electrolyte can easily regenerate all dyes. Dipole moment analysis was also performed. DSSCs based on the dyes, in both flavylium and quinoidal base forms, were assembled, and their photovoltaic performances were evaluated by measuring the open-circuit voltage, the short circuit current density, the fill factor, and the energy conversion efficiency. Results obtained by both experimental and computational studies showed that the overall performances of the DSSCs with the quinoidal forms were better than those obtained with the flavylium cations dyes.

## 1. Introduction

Dye-sensitized solar cells (DSSCs) attracted much attention in the late 1980s [1,2] due to many advantages, such as ease of fabrication, low cost, transparency, and flexibility. DSSCs have better performances than other solar cell technologies under diffuse light conditions and higher temperatures [3].

**Figure 1:**
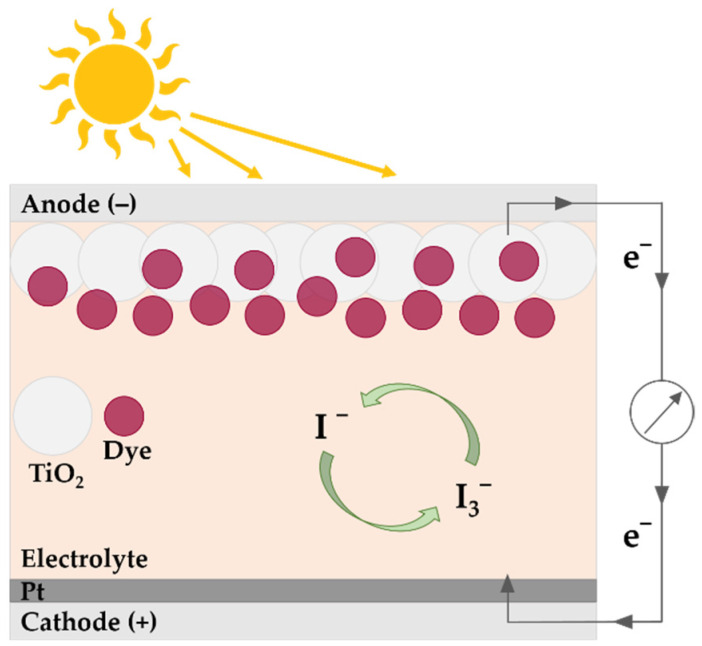
Schematic representation of a DSSC.

The DSSCs are usually assembled using a sandwich-type approach, having, in succession, an electrode with a porous layer of nanocrystalline wide band gap semiconductor, the most widely used is TiO_2_, covered with a sensitizing dye, a redox electrolyte, such as iodide–triiodide ($I^{-}/I_{3}^{-}$), and a counter electrode, with a catalyst (graphite or platinum particles) deposited on the inner surface (Figure 1). The electrodes are transparent conductive oxide (TCO) on glass [4]. The most widely investigated TCO for DSSC applications is the fluorine-doped tin oxide (FTO) [5].

In DSSCs the charge separation is obtained through the photoexcitation of the dye from the ground state D to the excited state D*. This leads to the injection of the electron into the conduction band of the semiconductor (TiO_2_) [6], passing through the electrode into the external circuit and finally to the catalyst at the counter electrode. The dye is regenerated by electron transfer from the electrolyte $I^{-}$ (which reduces the positively charged dye), while the triiodide ions are reduced at the counter electrode, and the cycle is finished [7].

The efficiency of the photovoltaic device is strongly influenced by the sensitizers used, thus making them key components in DSSCs. A dye must have the following properties to be an efficient sensitizer in DSSCs [8,9,10,11]:

1. the ability to bind strongly to the semiconductor through anchoring groups, typically carboxylic or hydroxyl groups;
2. wide and intense absorption in the visible or near-IR region;
3. excited-state energy level of the dye higher than the conduction band edge of the semiconductor placed in the photoanode;
4. the HOMO energy must be lower than the redox potential of the electrolyte;
5. good thermal, photochemical, and chemical stability in both ground and excited states.

Natural dyes, mostly from one of the three main families: chlorophylls, betalains, and anthocyanins, have been the subject of several studies that showed them as promising efficient photosensitizers [12,13,14,15].

**Figure 2:**
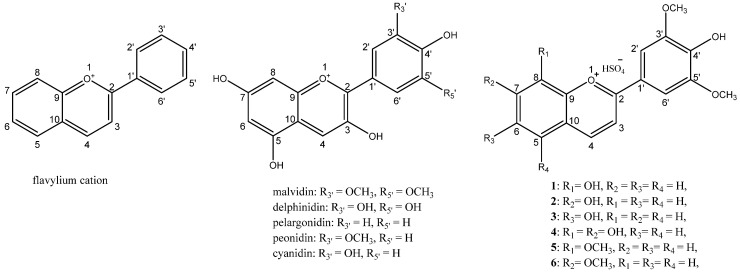
Structure of the flavylium cation, five natural and six (1–6) synthetic anthocyanidins.

Anthocyanins are versatile molecules belonging to the flavonoid group of phytochemicals. They are responsible for most red to blue colors exhibited by plants, including roots, stems, leaves, flowers, and fruits [16]. Their structures are based on a flavylium cation core (Figure 2), which is usually glycosylated in position 3 and sometimes 5 or less often in position 7 [17]. Due to their versatility, anthocyanins have numerous applications, ranging from coloring [18] to their antioxidant properties and other health benefits [19]. There is an increasing research interest in using them as multistate/multifunctional devices for information processing at the molecular level [20] and in photoelectronic technologies, such as solar cells [12,21]. The five most important aglycones of anthocyanins, anthocyanidins, are delphinidin, cyanidin, pelargonidin, malvidin, and peonidin (Figure 2).

Several reviews have studied the efficiency of natural dye-sensitized solar cells [22,23,24,25,26]. The overall conversion efficiencies (η) of solar cells sensitized with anthocyanins are mostly below 1%, ranging from 0.03% in the case of *Lithospermum* [27] to 1.5% in the case of *Sumac/Rhus* [28]. Many factors influence the performance and efficiency of DSSC. As for the natural dye sensitizers, the most important are: the methods and the solvents used for the extraction, the temperature of the extraction, the pH of the extract, and the interaction of the dye molecules with the TiO_2_ surface through anchoring groups responsible for the transfer of the excited electron from the sensitizer to the conduction band of the semiconductor.

**Figure 3:**
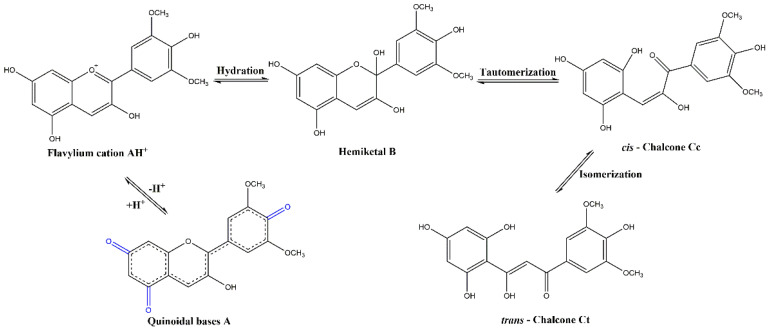
Structure of main malvidin species and their pH-dependent equilibria reactions.

The ability of the sensitizer to strongly bind to the TiO_2_ surface is of key importance, and in the case of anthocyanidins, there are three main possible binding modes: monodentate, chelate, and bridge bidentate [22,29]. The last two molecular interactions between the anthocyanidin and the TiO_2_ surface occur only if a cathecol fragment is present, in the case of cyanidin and delphinidin. Anthocyanidins exhibit several species in aqueous solutions depending on the pH (Figure 3); therefore, their binding modes to the TiO_2_ surface is influenced by the medium’s acidity.

The major drawbacks of natural dyes are their limited structural arrangements and their low stability upon contact with air and water [3,30,31].

To overcome these drawbacks, there is an increasing research interest in developing bio-inspired new synthetic compounds with tunable photoelectrochemical performances. In doing that, computational studies on both natural and synthetic compounds have provided insights into the physical reasons and the structure-related electronic factors responsible for the desired properties of a sensitizer for DSSCs [3,29,32,33,34].

New synthetic flavylium salts for DSSC purposes were obtained by introducing different electron donor/acceptor groups in the flavylium structure [3,32]. They were investigated by combined experimental and theoretical methods to understand how to design and synthesize flavylium compounds with improved photovoltaic performances. Both studies concluded that better performances are achieved by introducing strong donor groups, preferably in position 7.

We present a combined computational and experimental study involving spectroscopic, photophysical, and photoelectrochemical characterization of six new bio-inspired synthetic flavylium salts (Figure 2, compounds **1** to **6**) for DSSCs applications.

## 2. Materials and Methods

### 2.1. Materials and Methods

4′-hydroxy-3′,5′-dimethoxyacetophenone (97%), 2,3-dihydroxybenzaldehyde (97%), 2,4-dihydroxybenzaldehyde (98%), 2,5-dihydroxybenzaldehyde (98%), 2,3,4-trihydroxybenzaldehyde (98%), 2-hydroxy-3-methoxybenzaldehyde (98%), 2-hydroxy-4-methoxybenzaldehyde (98%), citric acid (>99.5%), boric acid (H_3_BO_3_, >99.5%), trisodium phosphate (Na_3_PO_4_, 96%) iodine (I_2_, 99.8%) and potassium iodide (KI, (>99%) were purchased from Sigma–Aldrich (Steinheim am Albuch, Germany). Sulfuric acid (H_2_SO_4_, 95–97%) and acetic acid (CH_3_COOH, 98%) were acquired from Merck KGaA (Darmstadt, Germany). Methanol (MeOH, >99%) and ethylene glycol (>99%) were purchased from CHIMREACTIV SRL (Bucuresti, Romania). HPLC grade solvents (methanol, acetonitrile) were used for the LC–MS measurements, and formic acid was of LC–MS grade; all were Sigma–Aldrich (Steinheim am Albuch, Germany).

All chemicals, reagents, and solvents were used without further purification.

The dye-sensitized solar cells were purchased from Solaronix (Aubonne, Switzerland) (Test Cell Kit 74991). The anode was made of FTO glass on which TiO_2_ was deposited; their active area was 0.36 cm^2^. The cathode was made of FTO glass with Pt deposited onto it.

#### 2.1.1. Synthesis of Flavylium Salts

The flavylium salts were obtained through a condensation reaction between 4′-hydroxy-3′,5′-dimethoxyacetophenone with the appropriate 2-hydroxybenzaldehyde (2,3-dihydroxybenzaldehyde; 2,4-dihydroxybenzaldehyde; 2,5-dihydroxybenzaldehyde; 2,3,4-trihydroxybenzaldehyde; 2-hydroxy-3-methoxybenzaldehyde; 2-hydroxy-4-methoxybenzaldehyde), by acid catalysis. The reactants were dissolved in a mixture of 12 mL acetic acid and 3 mL sulfuric acid, and stirred for 24 h at room temperature. In all cases, a precipitate was formed; it was filtered, washed with diethyl ether, and dried, yielding the hydrogensulfate flavylium salts.

#### 2.1.2. Halochromic Studies

The halochromic properties of the synthesized compounds were investigated through a UV–Vis spectroscopy study. According to a previously described procedure, buffer solutions in the range 2 to 12 were prepared from boric acid 0.2 M, citric acid 0.005M, and trisodium phosphate 0.1 M aqueous solutions [35]. The UV–Vis spectra of flavylium salts solutions (1.2 × 10^−4^ M to 5 × 10^−5^ M in methanol: water, see Supplementary Material Figures S25–S35) were registered in time at different pH values.

##### DSSC Photovoltaic Parameters

The anodes were immersed in dye solutions (1 mM) for 24 h in the dark at room temperature; afterward, they were washed with ethanol and left to dry. The redox electrolyte used was iodide/triiodide obtained according to a previously described procedure [36]. The cells were assembled in a sandwich-type manner.

The photovoltaic performance of the DSSC was evaluated based on four parameters: the open-circuit voltage (*V_OC_*), the short circuit current density (*J_SC_*), and the fill factor (*ff*, Equation (1)) [37] and the energy conversion efficiency (ECE or *η*, Equation (2)) [22].

(1) $$ff=\left(V_{max}\times J_{max}\right)/\left(V_{OC}\times J_{sc}\right)$$

where *V_max_* and *J_max_* are the respective voltage and current density values at maximum output power.

(2) $$\eta =\left(V_{OC}\times J_{sc}\times ff\right)/P_{in}$$

where *P_in_* is the input power (incident light) measured in mW∙cm^−2^.

These parameters were obtained from the photocurrent density-voltage (J–V) curve, where the current density is plotted against voltage when the DSSC is under standard AM 1.5 simulated sunlight (100 mW·cm^−2^).

### 2.2. Characterization of the Synthesized Flavylium Dyes

FT–IR spectra were obtained in attenuated total reflectance (ATR) mode on a Bruker Vertex 70 (Bruker Daltonik GmbH, Bremen, Germany) spectrometer equipped with a Platinium ATR, Bruker Diamond Type A225/Q. The spectra of the samples were collected on a spectral domain of 4000–400 cm^−1^, with a resolution of 4 cm^−1^ by co-addition of 64 scans.

NMR spectra were recorded on a Bruker AVANCE III spectrometer (Bruker Daltonik GmbH, Bremen, Germany) operating at 500.0 MHz (^1^H) and 125.0 MHz (^13^C) at 298 K. Chemical shifts δ are reported in ppm versus tetramethylsilane, TMS, coupling constants are reported in Hz, and the following abbreviations are used for splitting pattern: s (singlet), d (doublet), dd (doublet of doublets) and m (multiplet). For NMR assignments analysis of 1D NMR spectra (^1^H, ^13^C, DEPT 135) and 2D NMR spectra (COSY, HQSC, HMBC) have been performed. The samples were dissolved in DMSO-*d_6_*.

UV–Vis absorption spectra were recorded on an Agilent Cary 60 spectrophotometer at 20 °C (Agilent Technologies, Waldbronn, Germany).

LC–MS measurements were performed on an Agilent 1200 HPLC system coupled with Agilent 6410B triple Quadrupole Mass Spectrometer (Agilent Technologies, CA, USA), which was equipped with an electrospray ion source. The samples were dissolved in methanol, the separation was performed by isocratic elution using acetonitrile 40%/H_2_O and 60% formic acid 0.1% at a flow rate of 0.3 mL/min. The runtime was 7 min. The mass spectrometer was operated in positive ionization mode. The ion source was set to 350 °C; the capillary voltage was set to 4 kV, and the fragmentor voltage to 135 V. The results are given as [M]+, not as [M+H]+ as it is commonly when using ESI, because the compounds are already in ionic form.

A Keithley 2450 SourceMeter SMU recorded current^–^voltage curves using a home-made acquisition program. Instruments under simulated sunlight irradiation (AM 1.5 G simulated sunlight: 100 mW·cm^−2^).

The pH of solutions was measured a Mettler Toledo Seven Compact S210-K (Mettler Toledo, Columbus, OH, USA) at 25 °C.

Melting points were determined on a Carl Zeiss melting point apparatus (Carl Zeiss, Oberkochen, Germany) and are uncorrected.

8-hydroxy-4′-hydroxy-3′,5′-dimethoxyflavylium hydrogensulfate (1)

767.2 mg of burgundy precipitate, *η* = 77.5%, m.p. = 224–227 °C

FT–IR (ATR) cm^−1^: 3082; 2839; 1589; 1554; 1510; 1466; 1435; 1348; 1281; 1234; 1148; 1113; 1009; 933; 835; 777; 743; 673; 563; 476; 442.

^1^H-NMR (500 MHz, DMSO-*d_6_*, δ ppm): 3.97 (s, 6H); 7.62-7.64 (m, 3H); 7.85 (s, 2H); 8.91 (d, 1H, *J* = 9.24 Hz); 9.25 (d, 1H, *J* = 9.24 Hz).

^13^C-NMR (125 MHz, DMSO-*d_6_*, δ ppm): 56.6; 108.8; 118.0; 118.3; 119.7; 122.7; 124.7; 129.5; 144.5; 146.5; 148.5; 149.0; 152.9; 172.6.

LC–MS molecular formula C_17_H_15_O_5_^+^ requires calcd. 299.0914, found [M]^+^:299.00.

7-hydroxy-4′-hydroxy-3′,5′-dimethoxyflavylium hydrogensulfate (2)

723.6 mg of red-orange precipitate, η = 73.1%, m.p. = 226–229 °C

FT–IR (ATR) cm^−1^: 3082; 2980; 2893; 1630; 1576; 1539; 1458; 1346; 1223; 1140; 1111; 1061; 851; 577; 432.

^1^H-NMR (500 MHz, DMSO-*d_6_*, δ ppm): 3.98 (s, 6H); 7.41 (dd, 1H, *J* = 8.95, 2.2 Hz); 7.66 (d, 1H, *J* = 2.2 Hz); 7.80 (s, 2H); 8.19 (d, 1H, *J* = 8.95 Hz); 8.65 (d, 1H, *J* = 8.85 Hz); 9.20 (d, 1H, *J* = 8.85 Hz).

^13^C-NMR (125 MHz, DMSO-*d_6_*, δ ppm): 56.7; 103.0; 107.8; 113.2; 118.4; 118.6; 120.9; 132.6; 145.9; 148.8; 152.7; 158.3; 168.0; 171.0.

LC–MS molecular formula C_17_H_15_O_5_^+^ requires calcd. 299.0914, found [M]^+^: 299.10.

6-hydroxy-4′-hydroxy-3′,5′-dimethoxyflavylium hydrogensulfate (3)

900 mg of brown precipitate, η = 90.9%, m.p. = 207–210 °C

FT–IR (ATR) cm^−1^: 3354; 1601; 1547; 1499; 1458; 1342; 1306; 1271; 1194; 1109; 1043; 945; 862; 579; 473.

^1^H-NMR (500 MHz, DMSO-*d_6_*, δ ppm): 3.98 (s, 6H); 7.49 (d, 1H, *J* = 2.90 Hz); 7.72 (dd, 1H, *J* = 9.29, 2.90 Hz); 7.86 (s, 2H); 8.33 (d, 1H, *J* = 9.30 Hz); 8.90 (d, 1H, *J* = 9.32 Hz); 9.21 (d, 1H, *J* = 9.32 Hz).

^13^C-NMR (125 MHz, DMSO-*d_6_*, δ ppm): 56.8; 108.4; 111.2; 117.8; 118.5; 120.6; 125.1; 128.3; 147.1; 149.0; 149.8; 151.9; 157.8; 171.6.

LC–MS molecular formula C_17_H_15_O_5_^+^ requires calcd. 299.0914, found [M]^+^: 299.10.

7,8-dihydroxy-4′-hydroxy-3′,5′-dimethoxyflavylium hydrogensulfate (4)

844.6 mg of bright red precipitate, η = 82%, m.p. = 187–190 °C

FT–IR (ATR) cm^−1^: 3174; 2980; 1765; 1624; 1587; 1539; 1500; 1435; 1346; 1263; 1176; 1136; 1090; 1040; 847; 806; 758; 704; 573; 434.

^1^H-NMR (500 MHz, DMSO-*d_6_*, δ ppm): 3.97 (s, 6H); 7.46 (d, 1H, *J* = 8.84 Hz); 7.74 (d, 1H, *J* = 8.84 Hz); 7.84 (s, 2H); 8.62 (d, 1H, *J* = 8.90 Hz); 9.17 (d, 1H, *J* = 8.90 Hz).

^13^C-NMR (125 MHz, DMSO-*d_6_*, δ ppm): 56.7; 108.0; 112.9; 118.8; 119.0; 119.9; 122.7; 132.9; 145.9; 146.3; 148.8; 153.3; 156.4; 170.9.

LC–MS molecular formula C_17_H_15_O_6_^+^ requires calcd. 315.0863, found [M]^+^: 315.10.

8-methoxy-4′-hydroxy-3′,5′-dimethoxyflavylium hydrogensulfate (5)

946.6 mg of dark purple precipitate, η = 92.35%, m.p. = 234–237 °C

FT–IR (ATR) cm^−1^: 3076; 2978; 1591; 1549; 1497; 1431; 1350; 1273; 1178; 1101; 1043; 872; 741; 565; 482; 430.

^1^H-NMR (500 MHz, DMSO-*d_6_*, δ ppm): 3.96 (s, 6H); 4.14 (s, 3H); 7.80-7.76 (m, 5H); 8.93 (d, 1H, *J* = 9.33 Hz); 9.22 (d, 1H, *J* = 9.33 Hz).

^13^C-NMR (125 MHz, DMSO-*d_6_*, δ ppm): 56.5; 57.1; 108.4; 117.8; 118.3; 120.5; 124.2; 129.2; 144.6; 148.0; 149.2; 149.7; 151.9; 158.1; 171.7.

LC–MS molecular formula C_18_H_17_O_5_^+^ requires calcd. 313.1071, found [M]^+^: 313.10.

7-methoxy-4′-hydroxy-3′,5′-dimethoxyflavylium hydrogensulfate (6)

866 mg of dark red precipitate, η = 84.5%, m.p. = 224–227 °C

FT–IR (ATR) cm^−1^: 3086; 2978; 1628; 1578; 1456; 1342; 1219; 1140; 1105; 1009; 851; 569; 469; 413.

^1^H-NMR (500 MHz, DMSO-*d_6_*, δ ppm): 3.99 (s, 6H); 4.12 (s, 3H); 7.52 (d, 1H, *J* = 10.6 Hz); 7.84 (s, 2H); 8.04 (s, 1H); 8.22 (d, 1H, *J* = 9.0 Hz); 8.75 (d, 1H, *J* = 8.9 Hz); 9.24 (d, 1H, *J* = 8.9 Hz).

^13^C-NMR (125 MHz, DMSO-*d_6_*, δ ppm): 56.8; 57.4; 101.0; 108.2; 114.5; 118.4; 118.9; 120.6; 131.7; 146.6; 148.9; 152.7; 158.1; 167.8; 171.6.

LC–MS molecular formula C_18_H_17_O_5_^+^ requires calcd. 313.1071, found [M]^+^: 313.10.

### 2.3. Computational Studies

All computational studies were performed using the Gaussian 09, Revision B01 program package [38], and the density functional theory (DFT) methods at the B3LYP/6-31+G (d, p) level of theory [39].

Geometry optimization and frequency calculations were performed on all flavylium salts structures in the solvent phase (methanol) using the polarizable continuum model (PCM) with the integral equation formalism variant (IEFPCM) [40,41,42].

The electronic absorption spectra were computed in methanol with the IEFPCM model by time-dependent DFT (TD–DFT) calculations on the optimized structures at the B3LYP/6-31+G (d, p) level of theory. The lowest six singlet→singlet spins allowed excited states were taken into account.

## 3. Results and Discussions

### 3.1. Computational Studies

#### 3.1.1. Geometry Optimization and Frequency Calculations

**Figure 4:**
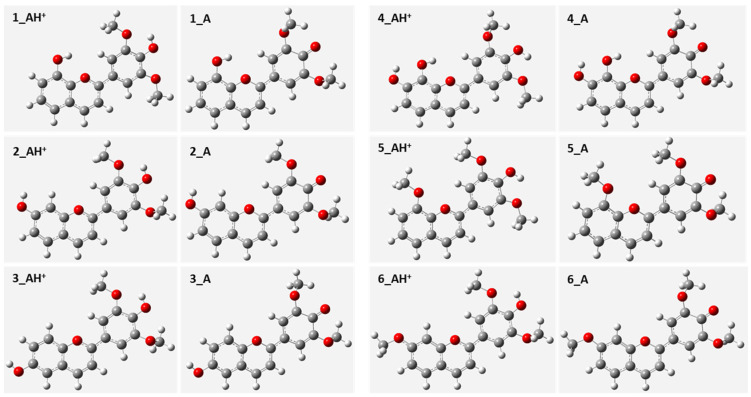
Optimized structures of the dyes (flavylium salts AH^+^ and quinoidal bases A).

Geometry optimization and frequency calculations were performed on all structures at the B3LYP/6-31+G (d, p) level of theory. The obtained structures (Figure 4) were energy minima (with no imaginary frequencies).

All investigated dyes, regardless of the specie, flavylium cations or quinoidal bases, present a planar geometry consistent with the extended π conjugation involving the substituted benzopyrylium and the substituted benzene moiety from malvidin (4′-hydroxy-3′, 5′-dimethoxybenzene).

#### 3.1.2. Energetic Parameters and Density Functional Theory-Based Reactivity Descriptors

Conceptual DFT reactivity descriptors offer important information on chemical reactivity and stability. Among them, the most used ones are electronegativity (*χ*), chemical potential (*µ*), absolute chemical hardness (*η*), electrophilicity (*ω*), as well as the HOMO-LUMO energy gap (Δ*E*). All these descriptors were calculated based on Equations (3–6) [43].

(3) $$\chi =-\mu =-(E_{HOMO}+E_{LUMO})/2$$

(4) $$\eta =(E_{LUMO}-E_{HOMO})/2$$

(5) $$\omega =\mu^{2}/2\eta$$

(6) $$\Delta E=E_{LUMO}-E_{HOMO}$$

where *E_HOMO_* is the energy of the highest occupied molecular orbital and *E_LUMO_* is the energy of the lowest unoccupied molecular orbital.

Table 1 summarizes the energetic parameters and the reactivity descriptors within the conceptual DFT calculated for the flavylium dyes at the B3LYP/6-31+G (d, p) level of theory.

An important requirement for dyes to be suitable for DSSC applications is that the LUMO level should be above the conduction band edge of the semiconductor (TiO_2_) for the electron to be effectively injected, and the HOMO level should be below the redox potential of the electrolyte ($I^{-}/I_{3}^{-}$) to ensure that the oxidized dye molecules can be efficiently regenerated. Another important aspect is for the dyes to have a narrow enough HOMO–LUMO energy gap to shift the absorption into the visible region and a distinct orbital localization to ensure charge separation or directionality.

From Table 1, there are three evident trends, HOMO and LUMO for the flavylium specie, AH^+^, which are lower in energy than those of the quinoidal bases, A; the difference is around 1 eV for HOMO and 0.8 eV for LUMO. The HOMO–LUMO energy gaps for the quinoidal specie are narrower than those for the flavylium cations, AH^+^ by approximately 2.5 eV. The absolute hardness and the HOMO-LUMO energy gap descriptors are related to the molecule’s stability. Their calculated values enlisted in Table 1 show that the flavylium cation forms are more stable than the quinoidal bases for all dyes. A lower chemical hardness is desired for photovoltaic applications, resulting in lower intramolecular charge transfer resistance. This leads to better short-circuit density and energy conversion efficiency [44,45]. Therefore, the quinoidal base species should exhibit better short-current densities than the flavylium cations.

**Figure 5:**
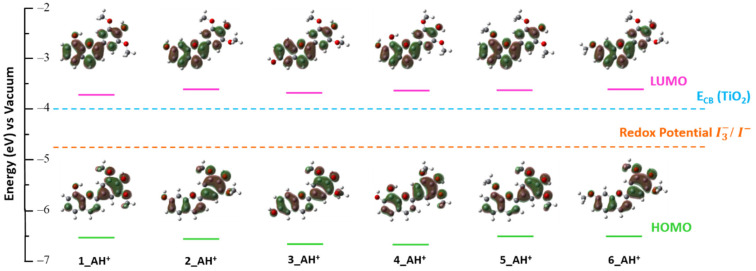
Energy level diagram for the dyes (flavylium cations AH^+^).

**Figure 6:**
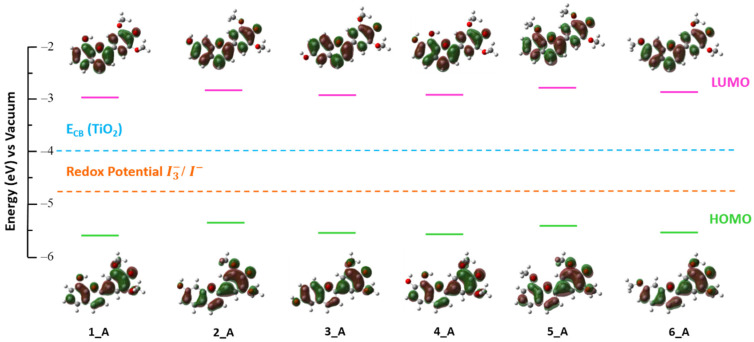
Energy level diagram for the dyes (quinoidal bases A).

Figure 5 and Figure 6 depict the energy level diagrams for the dyes in both flavylium and quinoidal forms, along with the plotted HOMO and LUMO molecular orbitals. The molecular orbital analysis shows a broad delocalization of both frontier orbitals on the flavylium structure with most substituents contribution. Although the HOMO distribution is similar to LUMO, for HOMO orbitals, it can be observed that the benzene fragment from malvidin has a greater contribution to the MO density, while for LUMO orbitals, this occurs in the benzopyrylium moiety.

Table 1 and Figure 5 and Figure 6 show that all dyes have the LUMO level higher than the conduction band of TiO_2_, for which the value used was E_CB_ = −4.0 eV (experimentally determined value in aqueous redox electrolytes [46]). Thus, they can inject electrons into the conduction band of the TiO_2_ upon excitation and the HOMO level below the iodide/triiodide redox potential for which the value used was E_I_^−^_/I3_^−^_ =_ −4.8 eV [47], indicating that the redox couple can regenerate the oxidized dyes.

From Figure 5, it can be observed that in the flavylium specie, the LUMO orbitals are closer to the conduction band of TiO_2,_ which could suggest a faster/more favorable electron injection into the semiconductor band. The HOMO orbitals are much lower than the iodide/triiodide redox potential, indicating the more energy-consuming regeneration process of the oxidized dyes. Another noticeable aspect is that in the case of LUMO orbitals, the substituents in positions R_3’_ and R_5’_ do not contribute to the MO density. In the case of dye 3, there is no contribution from the substituent in position R_6_. This could be an indicator that the electron injection should take place from the substituents which contribute to LUMO density, namely R_8_ and R_4’_ for dye 1; R_7_ and R_4’_ for dyes 2, 5, and 6; R_4’_ for dye 3; R_7,_ R_8,_ and R_4’_ for dye 4.

Figure 6 reveals that the situation is reversed in the case of the quinoidal base specie. The energy gap between the HOMO orbitals and the conduction band of TiO_2_ is wider, which can be an indicator of slower electron injection into the semiconductor band. On the other hand, the gap between the LUMO orbitals and the iodide/triiodide redox potential is narrower, suggesting a faster regeneration of the oxidized dyes by the redox couple. The MO analysis for the quinoidal base species revealed that the substituents which do not contribute to the LUMO density are in position R_5’_ for all dyes; for dyes 1, 3, 4, and 6, there is no contribution from the substituent in position R_3’_ also, for dye 3 and 6 it is barely noticeable. As with the situation for the flavylium form of dye 3, the substituent in position R_6_ of the quinoidal base specie does not contribute to the MO density. This would suggest that the electron injection should occur from the substituents which contribute to LUMO density, namely: R_8_ and R_4’_ for dye 1; R_7_, R_3’_ and R_4’_ for dyes 2 and 5; R_4’_ for dye 3; R_7,_ R_8_ and R_4’_ for dye 4; R_7_ and R_4’_ for dye 6.

#### 3.1.3. Computed Electronic Absorption Spectra

Time-dependent calculations (TD–DFT) were performed on the ground state geometry optimized structures of both flavylium and quinoidal base forms considering the solvation effect (methanol) to obtain the vertical excitation energies. The calculated excitation energies, E (eV), oscillator strength, f, absorption wavelength, λ (nm), light harvesting efficiency, LHE, for the first vertical transition, and the excited state lifetime for the dyes are listed in Table 2.

The simulated data show that the dominant electronic transitions for all considered structures are from HOMO to LUMO with oscillator strengths between 0.2 and 1.1.

Light-harvesting efficiency is an important parameter for the efficiency of DSSCs, related to the dye’s response to incident light, and can be determined from Equation (7) [12,48,49,50].

(7) $$LHE=1-10^{-f}$$

where f is the oscillator strength of the dye corresponding to the maximum absorption.

The higher the values for LHE are, the greater the photocurrent response is, which leads to better performances of the dyes as sensitizers for DSSCs. As a trend, all flavylium cations present smaller oscillator strengths than those of the quinoidal bases, thus having lower light-harvesting efficiencies.

The best results of LHE are obtained for the quinoidal species of dye 6, followed by dyes 4 and 2 with almost the same LHE. The results obtained for the flavylium cations suggest that the best performances in terms of photocurrent response should show dyes 6 and 2.

The excited-state lifetime *τ* is an important dye property that can estimate the electron injection efficiency into the semiconductor. A short electron lifetime could result from recombination processes leading to reduced charge collection efficiency, decreased short circuit photocurrent density, and photocurrent efficiency. A dye with a longer excited-state lifetime should be more suitable for easier charge transfer. The excited-state lifetime can be evaluated by Equation (8) [45,51,52].

(8) $$\tau =1.499/f\times E^{2}$$

where f is the f is the oscillator strength of the electronic state and E is the excitation energy of different electronic states (cm^−1^).

#### 3.1.4. Computed Photovoltaic Parameters

Computational methods can evaluate the DSSCs performances by determining different parameters. This study aimed to determine the most important and refereed six parameters. One of the most important parameters for DSSCs performance determinations is the IPCE, incident photon to electron conversion efficiency. The IPCE can be obtained as a product of light-harvesting efficiency (*LHE*), electron injection efficiency (*θ_inject_*), and charge collection efficiency (*η_c_*) and can be determined by Equation (9) [53,54,55,56].

(9) $$IPCE=LHE\times \theta_{inject}\times \eta_{c}$$

The electron injection efficiency (*θ_inject_*) is related to the free energy change of electron injection, Δ*G^inject^*, from dyes to the semiconductor surface and can be expressed as in Equation (10) [33,57,58,59,60].

(10) $$\Delta G^{inject}=E_{OX}^{dye*}-E_{CB}^{TiO_{2}}$$

where $E_{OX}^{dye*}$ is the oxidation potential of the dye in the excited state, given in Equation (11) [33,58,59], and $E_{CB}^{TiO_{2}}$ is the band edge of the titanium oxide conduction band.

(11) $$E_{OX}^{dye*}=E_{OX}^{dye}-\lambda_{max}^{ICT}=E_{OX}^{dye}-\lambda_{0-0}^{dye}$$

where $E_{OX}^{dye}$ is the oxidation potential of the dye in the ground state and is related to the *E_HOMO_* and $\lambda_{max}^{ICT}$ is the energy of the photoinduced intramolecular charge transfer, which can be considered as the transition energy between the ground state and the excited state, $\lambda_{0-0}^{dye}$, also referred to as the excitation energy, *E* (eV).

The dye regeneration is another factor that influences the performance of DSSCs. It can be evaluated by calculating the free energy change regeneration Δ*G^regen^* as given in Equation (12) [56].

(12) $$\Delta G^{regen}=E_{OX}^{dye}-E_{redox}^{electrolyte}$$

where $E_{redox}^{electrolyte}$ is the redox potential of the electrolyte, in this case, is the iodide/triiodide.

The open-circuit voltage, *V_OC_*, is a key parameter in determining the efficiency of DSSCs. It is calculated as the difference between the quasi-Fermi level of the semiconductor and the electrolyte redox potential [33]. However, it can be approximately evaluated as Equation (13) [58].

(13) $$V_{OC}=E_{LUMO}-E_{CB}^{TiO_{2}}$$

where *E_LUMO_* is the energy of the lowest unoccupied molecular orbital and $E_{CB}^{{TiO}_{2}}$ is the energy of the band edge of the titanium oxide conduction band.

All the above-defined parameters were calculated for the investigated dyes, and the results are presented in Table 3.

The light-harvesting efficiency (LHE), the open-circuit voltage (*V_OC_*), the free energy change of electron injection (Δ*G^inject^*) and the free energy change regeneration (Δ*G^regen^*) are the most important parameters in the performance evaluation of DSSCs because they are related to the properties that a dye must have to be a good sensitizer. LHE is indicative of absorption. *V_OC_* and Δ*G^inject^* can be associated with the dye’s ability to efficiently inject electrons into the semiconductor conduction band, while Δ*G^regen^* to the regeneration of the oxidized dye by electron transfer from the electrolyte.

The Δ*G^inject^* values reflect the ease with which electrons can be injected into the semiconductor conduction band, thus the electron injection efficiency. The more negative the values of Δ*G^inject^* are, the more favorable the electron injection into the TiO_2_ surface is expected to be. All the investigated dyes have been found to inject electrons into the semiconductor conduction band because of their negative Δ*G^inject^* values. Based on the results, the quinoidal bases should inject more efficiently electrons into the semiconductor than the flavylium cations. The best results for the flavylium species were found for dye 2, closely followed by dye 6 and 5. Quinoidal bases dye 2 exhibited the best electron injection efficiency, followed by dyes 5 and 6.

Positive values for the free energy change regeneration (ΔG^regen^) indicate that the electrolyte can be regenerated through electron transfer. Larger ΔG^regen^ values imply faster regeneration processes and less electron recombination. The results obtained show that the redox electrolyte can easily regenerate all dyes. The values of ΔG^regen^ for the quinoidal bases are higher, suggesting that they are faster/easier regenerated than the flavylium cation. In both series, the values are within 5% differences, thus having a very small influence on the performances of the DSSCs.

In the case of the V_OC_ calculated values, the best results were obtained for quinoidal base forms of the dyes, with values up to five times larger than those obtained for the flavylium cations. Dyes 6, closely followed by dyes 2 and 5 as flavylium specie, exhibited the best open-circuit voltages in their series, while the quinoidal forms of dyes 2, followed by dyes 5 and 6, showed the best photovoltaic performances according to the V_OC_ parameter.

#### 3.1.5. Dipole Moment Analysis

Dipole moments influence several processes related to photovoltaic performances: the electron injection, the V_OC_, and recombination. Large dipole moments lead to increased electron injection. The photocurrent generation is associated with the intramolecular charge transfer, evidenced by larger dipole moments in the excited state than in the ground state [61,62].

It has been well established that the dipole moment change (∆µ*_ge_*), which can be calculated using Equation (14), correlates linearly with photovoltaic properties [63]. To gain additional information on the role of different substituents and their position on photovoltaic performances, we calculated the ∆µ*_ge_* from DFT calculations (Table 4).

(14) Δµge=[(µgx−µex)2+(µgy−µey)2+(µgz−µez)2]12

Large ∆µ*_ge_* values, associated with enhanced polarizability, enable appropriate charge generation processes [64,65].

The results presented in Table 4 show that the dipole moments values in the excited states are greater for all dyes than those in ground states, which indicates an intramolecular charge transfer.

It can also be observed that the quinoidal base species have much higher values of ∆µ_ge_ than the flavylium cations for all considered dyes; as a result/consequence, the quinoidal base species should exhibit more favorable charge generation and easier charge separation. The largest values of ∆µ_ge_ were obtained for dye 5 for both species, flavylium cation and quinoidal base, followed by dye 6 in the case of AH^+^ forms and by dye 2 in the case of A forms, for which the value was almost equal to that obtained for dye 6.

From the computational studies performed, dye 6, followed by dyes 2 and 5, are the best candidates as sensitizers for DSSC. The best results from computations show that better photovoltaic performances are obtained for compounds containing strong donor groups (–OCH_3_ > –OH), preferably in position 7 over 8.

### 3.2. Synthesis and Characterization of The Flavylium Salts

For DSSC applications, the research on synthesizing new flavylium analogs is continuously increasing because the photoelectrochemical performances of these dyes can be significantly improved through rational design. Introducing new functional groups or substituting others can lead to tailored energy levels, better absorption properties, and greater charge transfer to the semiconductor.

**Scheme 1:**
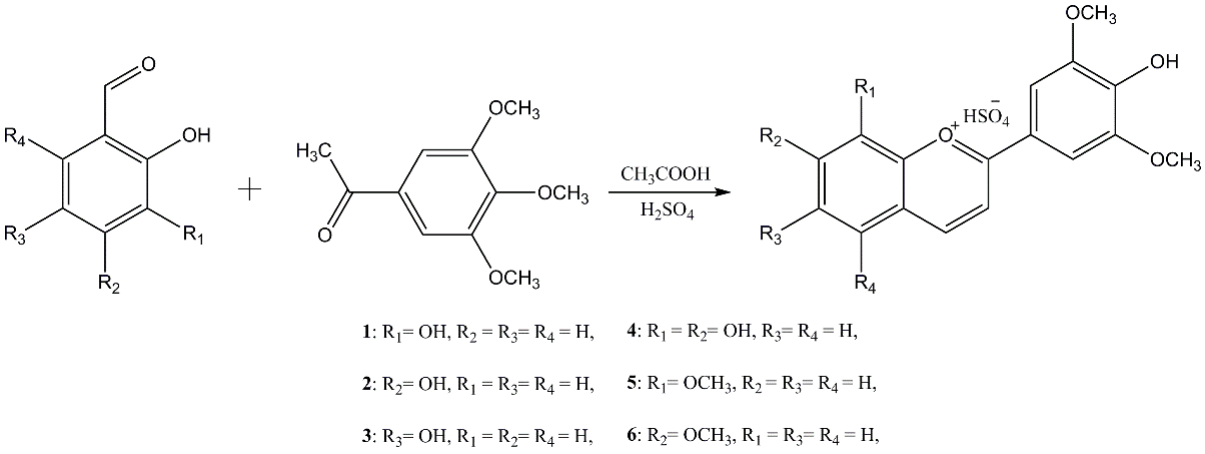
Reaction scheme for the synthesis of the flavylium dyes.

The flavylium analogs were synthesized through a sustainable synthetic route by acid-catalyzed condensation between acetophenones and substituted salicylaldehydes (Scheme 1).

Their structural identities were demonstrated by FT–IR and LC–MS methods (Supplementary Material, Figures S13–S24). Their structures and purities were confirmed by NMR analysis (Supplementary Material, Figures S1–S12). The main characterization parameters of the synthesized flavylium dyes are presented in the Supplementary Material, Table S1.

In Table 5, the experimental maximum absorption wavelengths, and the absorption energies of the flavylium dyes are presented. These results concord with the theoretical data from computational studies included in Table 2.

### 3.3. Halochromic Properties Evaluation

**Figure 7:**
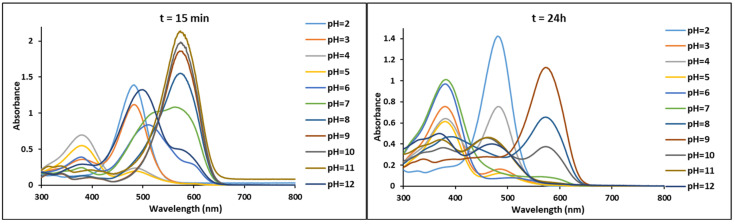
UV–Vis spectra of dye 2 in different pH buffer solutions (3.8 × 10^−5^ M in methanol: water 1:9).

Different groups demonstrated the halochromic behavior of the flavylium derivatives. For all six synthesized compounds, the halochromic properties were evaluated spectrophotometrically. Upon pH shifts, color changes were observed, and the overlaid collected UV–Vis spectra for dye 2 are presented in Figure 7. This proved the existence of multiple species at different pH values. All collected UV–Vis spectra for the other five flavylium dyes are presented in Figures S25–S35 (Supplementary Material).

**Scheme 2:**
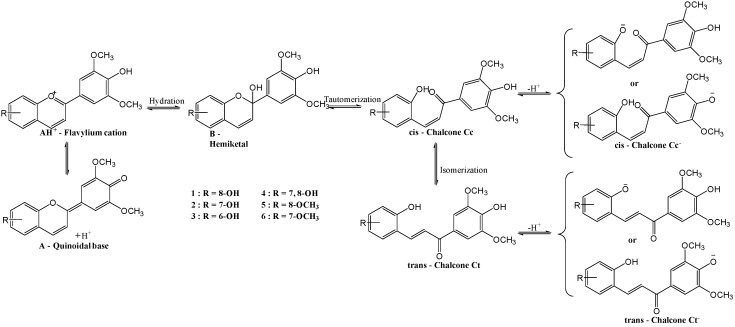
The network of chemical reactions of the flavylium dyes upon pH change.

The available reports on pH transformations of flavylium derivatives, whether natural or synthetic, follow the same network of reactions upon pH change [21]. The proposed network of chemical reactions upon pH change for the derivatives synthesized in this study is presented in Scheme 2. The chemical equilibrium reaction network gives rise to several species, stable at different pH values. At very low pH values (pH ≤ 3), the red flavylium cation AH^+^ is the stable species. With the pH value increasing (3 to 7), the AH^+^ species undergoes two competing transformations: into the blue quinoidal base A by a fast proton transfer process and into the colorless or pale yellow hemiketal B by a slow hydration process. The hemiketal turns into the *cis*-chalcone Cc by a tautomerization process, which is subsequently transformed into the *trans*-chalcone Ct by a slow isomerization process. Both light and pH stimulation can achieve *cis-* and *trans*-chalcone interconversion. Anionic species can be formed in basic conditions by deprotonation (A^−^, Cc^n−^, Ct^n^).

Based on the UV–Vis spectra collected in time, the stability of the species at different pH values was evaluated, and the results are presented in Table 6.

It can be observed that the AH^+^ species are stable in time at pH values lower than 3, and Ct^n−^ species are stable in time at pH values ≥ 11. The other species, A, Cc, and Ct, are not stable, and a decrease in the color intensity leading up to colorless solutions was observed in time, probably due to the slower hydration process that favors the formation of the B species.

The UV–Vis spectroscopy study performed in time at different pH values evidenced the pH-dependent conversion between the species involved in the network of chemical reactions (Supplementary Material, Figure S30–S35).

### 3.4. Photovoltaic Performances of DSSCs

**Figure 8:**
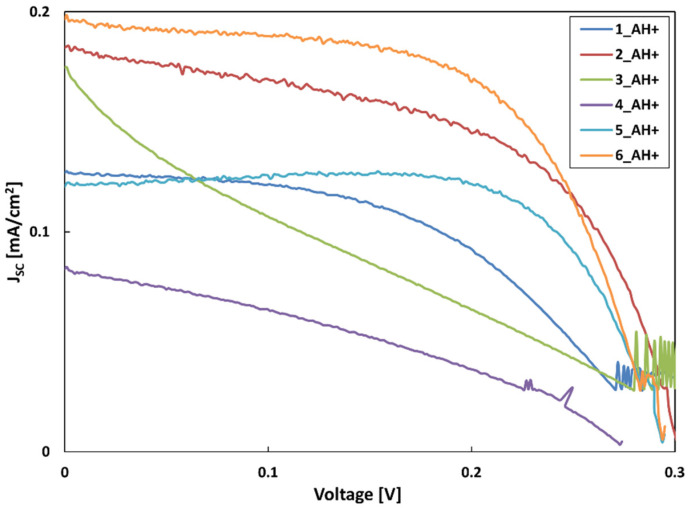
The J–V curves of DSSCs based on the flavylium cations AH^+^ under 100 mW·cm^−2^ simulated AM 1.5 G illumination.

The photovoltaic performances of the DSSCs sensitized with the flavylium dyes were evaluated by measuring the key parameters (short circuit current density (*J_SC_*), open-circuit voltage (*V_OC_*), fill factor (*ff*), and the overall conversion efficiency (*η*)). Figure 8 presents the current–voltage curves measured for the DSSCs with the flavylium dyes.

In terms of generated maximum power outputs, the best performances were obtained for the DSSCs sensitized with dye 6, followed by dyes 2 and 5, in the AH^+^ series. This may be explained because these dyes exhibited the best absorption properties, as shown by the computed LHE values. Moreover, they can easily inject electrons into the TiO_2_ conduction band, as predicted by ΔG^inject^ calculated values. Concerning the chemical structures of the compounds, interesting and very useful information can be derived, namely a correlation between the nature and position of the substituent onto the flavylium moiety. First, the performances are directly correlated with the power of the donor group. The compounds bearing –OCH_3_ substituents were more effective than those presenting –OH groups in the same position. More precisely, compound 6 presents –OCH_3_ donor substituent in position 7, and compound 2 bears the –OH donor in the same position. The same tendency was observed for compounds 5 (–OCH_3_ donor) and 1 (–OH donor), where the substituent position is 8. Based on these results, we can affirm that strong donor groups in position 7 lead to more favorable photovoltaic performances.

Moreover, these experimental data are in concordance with the theoretical results described in Section 3.1.4. and confirm the applicability and usefulness of these theoretical studies to design molecules with specific photovoltaic properties. The results obtained for compound 3 reinforce the previous statements because compound 3 bears –OH donor substituent, and the performance was lower than the compounds 1 and 2 because the substituent position is 6. The lowest J_SC_ and V_OC_ values were obtained for compound 4, which presents two –OH donor substituents in positions 7 and 8.

**Figure 9:**
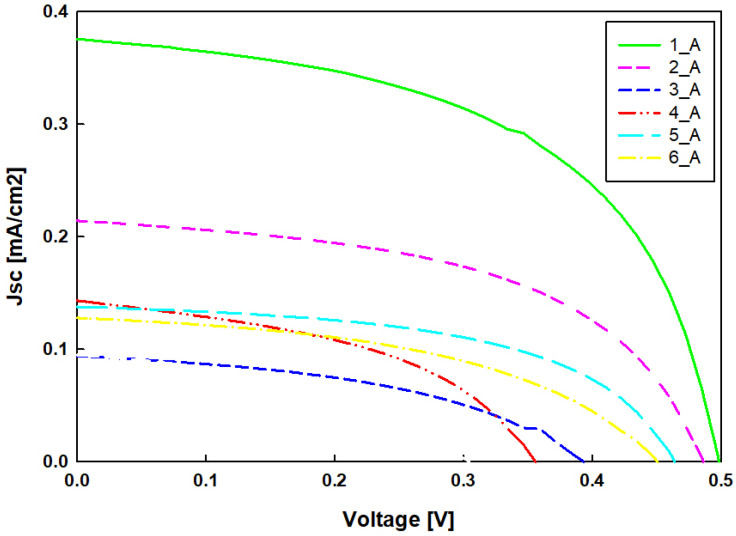
The J–V curves of DSSCs based on the quinoidal bases A under 100 mW·cm^−2^ simulated AM 1.5 G illumination.

Figure 9 presents the current–voltage curves measured for the DSSCs with the quinoidal bases.

The greatest generated maximum power outputs were obtained for the DSSCs sensitized with dye 1, followed by dye 2 and dyes 5 and 6, with close results when the dyes were in the quinoidal base form. The results for dyes 2, 5 and 6 correlate well with the absorption properties (LHE values) and the ΔG^inject^ calculated values, while for dye 1 no calculated parameter suggested it would have the best performance.

These results can be considered for the forward design of valuable molecules with photovoltaic properties.

All the other determined photovoltaic parameters for the DSSCs are presented in Table 7.

The most efficient DSSCs in energy conversion were sensitized with dyes 6, 2, and 5 in the AH^+^ serie. These results confirm the exact same order provided by the computational study. As for the DSSCs with dyes in the quinoidal form the most efficient were with dyes 1 and 2. Although the synthesized dyes were found to exhibit the necessary properties to be efficient sensitizers in DSSCs by theoretical calculation, the obtained efficiencies are low. However, there are several other factors that can impact the photovoltaic performances of a DSSC, such as decomposition, evaporation or bleaching of the electrolyte, ambient temperature and humidity, morphology of working substrate, which can result in cell instability and low efficiency.

Results obtained by both experimental and computational studies showed that the overall performances of the DSSCs with the quinoidal forms were better than those obtained with the flavylium cations dyes.

It must be emphasized that results of different groups can be truly compared only for measurements performed in the same conditions due to the several factors that strongly influence the photovoltaic performances of a DSSC, such as the TCO, the semiconductor, the dye, the redox electrolyte.

## 4. Conclusions

Six new flavylium dyes were synthesized following a sustainable bio-inspired strategy for DSSCs applications. The compounds were characterized by FT–IR, UV–Vis, and NMR spectroscopy methods and LC–MS spectrometry techniques. Their pH-dependent photochromic properties were investigated through a UV–Vis spectroscopy study. It was found that they follow the same network of chemical reactions and present the same species as their natural analogs, the anthocyanins.

A computational study was performed to evaluate their suitability as sensitizers for DSSCs (in both flavylium cation and quinoidal base forms) and better understand the relationship between their molecular structures and their electronic, photophysical, and photoelectrochemical properties. The study showed that all the dyes have LUMO and HOMO energies that would allow efficient electron injection into the semiconductor and the possibility of being regenerated by the redox electrolyte from the oxidized state back to the ground state. The calculated photovoltaic parameters further confirmed this essential condition for a sensitizer: the free energy change of electron injection ΔG^inject^ and the free energy change regeneration ΔG^regen^. Time-dependent calculations (TD–DFT) revealed that the predominant transition for all dyes is from HOMO to LUMO. The light-harvesting efficiency, a measure of the dye’s response to incident light, was calculated for all flavylium dyes. The excitation energies, E (eV), the oscillator strength, f, the absorption wavelength, λ (nm) were also computed. The results obtained by computational methods suggested dyes 6, 2 and 5, as best suitable for DSSC applications regardless of their form, flavylium cation or quinoidal base.

DSSCs based on the synthesized dyes were assembled and their photovoltaic parameters were measured. The best results in the AH^+^ serie were obtained with dyes 6, 2, and 5 as sensitizers, thus confirming the theoretical findings. Better overall performances of the DSSCs were obtained with quinoidal base forms as sensitizers, among which the best results were with dyes 1 and 2.

Both computational and experimental studies suggest that a strong donor group, preferably in position 7, is essential for flavylium analogs to be efficient sensitizers.

New synthetic flavylium dyes that are quasi-natural, non-toxic, and environmentally friendly can be easily cost-effectively synthesized through rational design. Their electronic, photophysical, and photoelectrochemical properties can be tailored to improve the performances of photovoltaic devices.

## Figures and Tables

**Table 1:** Energetic parameters and reactivity descriptors of the flavylium dyes computed at the B3LYP/6-31+G (d, p) level of theory.

Compound	E (a.u.)	E_HOMO_ (ev)	E_LUMO_ (ev)	ΔE_LUMO-HOMO_ (eV)	η (eV)	µ (eV)	ω (eV)
1_AH^+^	−1032.540	−6.562	−3.760	2.802	1.401	−5.161	9.504
1_A	−1032.098	−5.668	−2.992	2.675	1.338	−4.330	7.008
2_AH^+^	−1032.546	−6.570	−3.603	2.967	1.484	−5.087	8.720
2_A	−1032.107	−5.405	−2.803	2.602	1.301	−4.104	6.473
3_AH^+^	−1032.542	−6.602	−3.702	2.900	1.450	−5.152	9.153
3_A	−1032.075	−5.558	−2.942	2.616	1.308	−4.250	6.904
4__AH^+^	−1107.764	−6.600	−3.673	2.927	1.463	−5.136	9.014
4_A	−1107.324	−5.586	−2.923	2.663	1.331	−4.255	6.797
5_AH^+^	−1071.815	−6.509	−3.663	2.845	1.423	−5.086	9.090
5_A	−1071.370	−5.463	−2.843	2.620	1.310	−4.153	6.582
6_AH^+^	−1071.819	−6.548	−3.600	2.948	1.474	−5.074	8.731
6_A	−1071.377	−5.531	−2.872	2.659	1.329	−4.201	6.639

**Table 2:** The calculated excitation energies, E (eV), the oscillator strength, f, the absorption wavelength, λ (nm), and light-harvesting efficiency, LHE.

Compound	Calculated Absorption Wavelength λ (nm)	Excitation Energies E (eV)	Oscillator Strengths (f)	Light-Harvesting Efficiency LHE	Excited State LIFETIME (τ) (ns)
1_AH^+^	482.1 (HOMO → LUMO)	2.571	0.336	0.539	10.375
1_A	472.5 (HOMO → LUMO)	2.624	0.858	0.861	3.900
2_AH^+^	456.6 (HOMO → LUMO)	2.715	0.731	0.814	4.276
2_A	482.1 (HOMO → LUMO)	2.572	0.995	0.899	3.501
3_AH^+^	465.1 (HOMO → LUMO)	2.666	0.627	0.764	5.170
3_A	487.9 (HOMO → LUMO)	2.541	0.841	0.856	4.243
4_AH^+^	476.3 (HOMO → LUMO)	2.603	0.547	0.716	6.217
4_A	476.1 (HOMO → LUMO)	2.604	1.005	0.901	3.381
5_AH^+^	474.7 (HOMO → LUMO)	2.612	0.203	0.373	16.637
5_A	480.7 (HOMO → LUMO)	2.579	0.818	0.848	4.235
6_AH^+^	462.4 (HOMO → LUMO)	2.681	0.791	0.838	4.053
6_A	478.3 (HOMO → LUMO)	2.592	1.067	0.914	3.214

**Table 3:** Photovoltaic parameters computed at B3LYP/6-31+G (d, p) level of theory.

Compound	$E_{ox}^{dye}\;\left(eV\right)$	*E* (eV)	$E_{ox}^{dye*}\;\left(eV\right)$	Δ*G^inject^* (eV)	$\Delta G_{dye}^{regen}\;\left(eV\right)$	*V_OC_* (eV)
1_AH^+^	6.562	2.571	3.991	−0.009	1.762	0.240
1_A	5.668	2.624	3.044	−0.956	0.868	1.008
2_AH^+^	6.570	2.715	3.855	−0.145	1.770	0.397
2_A	5.405	2.572	2.833	−1.167	0.605	1.197
3_AH^+^	6.602	2.666	3.936	−0.064	1.802	0.298
3_A	5.558	2.541	3.017	−0.983	0.758	1.058
4_AH^+^	6.600	2.603	3.997	−0.003	1.800	0.327
4_A	5.586	2.604	2.982	−1.018	0.786	1.077
5_AH^+^	6.509	2.612	3.897	−0.103	1.709	0.337
5_A	5.463	2.579	2.884	−1.116	0.663	1.157
6_AH^+^	6.548	2.681	3.867	−0.133	1.748	0.400
6_A	5.531	2.592	2.939	−1.061	0.731	1.128

**Table 4:** Dipole moments of dyes 1–6 from DFT calculations.

Compound	Dipole Moment Ground State (µ_g_) (Debye)	Dipole Moment Excited State (µ_e_) (Debye)	Dipole Moment Change (∆µ_ge_) (Debye)
1_AH^+^	6.316	6.392	0.076
1_A	13.229	13.430	0.201
2_AH^+^	7.130	7.191	0.062
2_A	15.541	15.789	0.249
3_AH^+^	7.511	7.577	0.067
3_A	15.271	15.472	0.205
4_AH^+^	7.556	7.632	0.076
4_A	12.641	12.842	0.201
5_AH^+^	8.591	8.694	0.103
5_A	17.265	17.514	0.254
6_AH^+^	8.990	9.067	0.080
6_A	18.401	18.647	0.247

**Table 5:** Experimental absorption wavelengths and energies of the flavylium dyes.

Compound	Absorption Wavelength λ (nm)	Absorption Energy (eV)
1	471	2.633
2	482	2.573
3	490	2.531
4	497	2.495
5	472	2.627
6	480	2.583

**Table 6:** Absorption wavelengths of the predominant species of the flavylium dye solutions at different pH values.

Compound	Absorption Wavelength λ (nm)			
	AH^+^ [pH Range]	A [pH Range]	Cc, Ct [pH Range]	Ct^n−^ [pH Range]
1	471 [≤3]	549 [4–10]	361, 396 [4–9, 10–11]	418 [≥12]
2	482 [≤3]	575 [7–11]	380, 390 [4–6, 10–11]	497 [≥12]
3	490 [≤3]	559 [4–10]	356, 398 [5–9, 10–11]	427 [≥11]
4	497 [≤2]	593 [7–11]	379, 392 [3–6, 10–11]	502 [≥12]
5	472 [≤3]	514 [4–10]	380, 393 [4–8, 9–10]	448 [≥11]
6	480 [≤3]	553 [7–10]	378, 440 [4–6, 9–10]	458 [≥11]

**Table 7:** The photovoltaic parameters of the DSSCs based on the flavylium dyes.

Compound	Jsc (mA∙cm^−2^)	Voc (mV)	Jmax (mA∙cm^−2^)	Vmax (mV)	ff	H (%)
1_AH^+^	0.127	326	0.096	193	0.449	0.052
1_A	0.375	488	0.276	365	0.550	0.279
2_AH^+^	0.184	302	0.135	226	0.550	0.085
2_A	0.214	478	0.155	348	0.529	0.150
3_AH^+^	0.175	339	0.049	301	0.251	0.041
3_A	0.093	391	0.063	256	0.442	0.045
4__AH^+^	0.083	274	0.045	176	0.350	0.022
4_A	0.143	352	0.094	242	0.453	0.063
5_AH^+^	0.121	295	0.114	223	0.713	0.071
5_A	0.073	468	0.051	324	0.483	0.046
6_AH^+^	0.197	295	0.164	211	0.596	0.096
6_A	0.104	430	0.070	277	0.437	0.054

## Data Availability

Not applicable.

## Supplementary Materials

The following supporting information can be downloaded at: https://www.mdpi.com/article/10.3390/ma15196985/s1, Figures S1–S12: ^1^H-NMR and ^13^C-NMR spectra of compounds 1–6; Figures S13–S24: Chromatograms and mass spectra of compounds 1–6; Figures S25–S29: UV–Vis spectra of compounds 1–6 in different pH buffer solutions; Figures S30–S35: UV–Vis spectra of compounds 1–6 at pH values from 2 to 12 in time, Table S1: The main characterization parameters of the synthesized flavylium dyes.

## References

[1] Desilvestro J., Gratzel M., Kavan L., Moser J., Augustynski J. (1985). Highly efficient sensitization of titanium dioxide. J. Am. Chem. Soc..

[2] Nazeeruddin M.K., Liska P., Moser J., Vlachopoulos N., Gratzel M. (1990). Conversion of Light into Electricity with Trinuclear Ruthenium Complexes Adsorbed on Textured TiO_2_ Films. Helv. Chim. Acta.

[3] Calogero G., Sinopoli A., Citro I., Di Marco G., Petrov V., Diniz A.M., Parola A.J., Pina F. (2013). Synthetic analogues of anthocyanins as sensitizers for dye-sensitized solar cells. Photochem. Photobiol. Sci..

[4] O’Regan B., Grätzel M. (1991). A low-cost, high-efficiency solar cell based on dye-sensitized colloidal TiO_2_ films. Nature.

[5] Lytle W.O., Junge A.E. (1951). Electroconductive Products and Production Thereof. U.S. Patent.

[6] Thorsmølle V.K., Wenger B., Teuscher J., Bauer C., Moser J.-E. (2007). Dynamics of Photoinduced Interfacial Electron Transfer and Charge Transport in Dye-Sensitized Mesoscopic Semiconductors. Photochem. Chim..

[7] Rowley J.G., Farnum B.H., Ardo S., Meyer G.J. (2010). Iodide Chemistry in Dye-Sensitized Solar Cells: Making and Breaking I−I Bonds for Solar Energy Conversion. J. Phys. Chem. Lett..

[8] Gratzel M. (2005). Solar Energy Conversion by Dye-Sensitized Photovoltaic Cells. Inorg. Chem..

[9] Ardo S., Meyer G.J. (2009). Photodriven heterogeneous charge transfer with transition-metal compounds anchored to TiO_2_ semiconductor surfaces. Chem. Soc. Rev..

[10] Devadiga D., Selvakumar M., Shetty P., Santosh M.S. (2021). Recent progress in dye sensitized solar cell materials and photo-supercapacitors: A review. J. Power Sources.

[11] Mariotti N., Bonomo M., Fagiolari L., Barbero N., Gerbaldi C., Bella F., Barolo C. (2020). Recent advances in eco-friendly and cost-effective materials towards sustainable dye-sensitized solar cells. Green Chem..

[12] Calogero G., Bartolotta A., Di Marco G., Di Cardo A., Bonaccorso F. (2015). Vegetable-based dye-sensitized solar cells. Chem. Soc. Rev..

[13] Castillo-Robles J.A., Rocha-Rangel E., Ramírez-de-León J.A., Caballero-Rico F.C., Armendáriz-Mireles E.N. (2021). Advances on Dye-Sensitized Solar Cells (DSSCs) Nanostructures and Natural Colorants: A Review. J. Compos. Sci..

[14] Mejica G.F.C., Unpaprom Y., Balakrishnan D., Dussadee N., Buochareon S., Ramaraj R. (2022). Anthocyanin pigment-based dye-sensitized solar cells with improved pH-dependent photovoltaic properties. Sustain. Energy Technol. Assess..

[15] Yuan Y., Wan C. (2022). Dual Application of Waste Grape Skin for Photosensitizers and Counter Electrodes of Dye-Sensitized Solar Cells. Nanomaterials.

[16] Konczak I., Zhang W. (2004). Anthocyanins—More Than Nature’s Colours. J. Biomed. Biotechnol..

[17] Takeoka G.R., Dao L.T., Hurst W.J. (2002). Anthocyanins. Methods of Analysis for Functional Food and Nutraceuticals.

[18] Melo M.J., Parola A.J., Lima J.C., Pina F., Somani P.R. (2010). Chromogenic materials based on 2-phenyl-1-benzopyrylium: From Anthocyanins to synthetic Flavylium compounds. Chromic Materials and Their Applications.

[19] Kong J.M., Chia L.S., Goh N.K., Chia T.F., Brouillard R. (2003). Analysis and biological activities of anthocyanins. Phytochemistry.

[20] Pina F., Maestri M., Balzani V., Feringa B.L. (2001). Multistate/Multifunctional Molecular Level Systems-Photochromic Flavylium Compounds. Molecular Switches.

[21] Pina F., Melo M.J., Laia C.A.T., Parola A.J., Lima J.C. (2012). Chemistry and applications of flavylium compounds: A handful of colours. Chem. Soc. Rev..

[22] Hug H., Bader M., Mair P., Glatzal T. (2014). Biophotovoltaics: Natural pigments in dye sensitized solar cells. Appl. Energy.

[23] Shalini S., Balasundara Prabhu R., Prasanna S., Mallick T.K., Senthilarasu S. (2015). Review on natural dye sensitized solar cells: Operation, materials and methods. Renew. Sustain. Energy Rev..

[24] Richhariya G., Kumara A., Tekasakul P., Gupta B. (2017). Natural dyes for dye sensitized solar cell: A review. Renew. Sustain. Energy Rev..

[25] Kumara N.T.R.N., Lim A., Lim C.M., Petra M.I., Ekanayake P. (2017). Recent progress and utilization of natural pigments in dye sensitized solar cells: A review. Renew. Sustain. Energy Rev..

[26] Jalali T., Arkian P., Golshan M., Jalali M., Osfouri S. (2020). Performance evaluation of natural native dyes as photosensitizer in dye-sensitized solar cells. Opt. Mater..

[27] Huizhi Z., Wu L., Gao Y., Ma T. (2011). Dye-sensitized solar cells using 20 natural dyes as sensitizers. J. Photochem. Photobiol. A Chem..

[28] Suhaimi S., Shahimin M.M., Alahmed Z.A., Chysky J., Reshak A.H. (2015). Materials for enhanced dye-sensitized solar cell performance: Electrochemical application. Int. J. Electrochem. Sci..

[29] Sinopoli A., Citro I., Calogero G., Bartolotta A. (2017). Combined experimental and DFT-TDDFT investigation on anthocyanidins for application in dye-sensitised solar cells. Dyes Pigm..

[30] Patrocínio A.O.T., Mizoguchi S.K., Paterno L.G., Garcia C.G., Murakami Iha N.Y. (2009). Efficient and low-cost devices for solar energy conversion: Efficiency and stability of some natural-dye-sensitized solar cells. Synth. Met..

[31] Kabir F., Manir S., Bhuiyan M.M.H., Aftab S., Ghanbari H., Hasani A., Fawzy M., De Silva G.L.T., Mohammadzadeh M.R., Ahmadi R. (2022). Instability of dye-sensitized solar cells using natural dyes and approaches to improving stability—An overview. Sustain. Energy Technol. Assess..

[32] Calogero G., Citro I., Di Marco G., Caramori S., Casarin L., Bignozzi C.A., Avo J., Parola A.J., Pina F. (2017). Electronic and charge transfer properties of bio-inspired flavylium ions for applications in TiO_2_-based dye-sensitized solar cells. Photochem. Photobiol..

[33] Prima E.C., Hidayat N.N., Yuliarto B., Suyatman, Dipojono H.K. (2017). A combined spectroscopic and TDDFT study of natural dyes extracted from fruit peels of Citrus reticulata and Musa acuminata for dye-sensitized solar cells. Spectrochim. Acta A.

[34] Shi X., Li Y., Wang L. (2022). Two novel mono-hydroxyl pyranoanthocyanidins bearing dimethylamino substituent(s) for dye-sensitized solar cell. J. Mol. Struct..

[35] Carmody W.R. (1961). Easily prepared wide range buffer series. J. Chem. Educ..

[36] Ursu D., Vajda M., Miclau M. (2019). Investigation of the p-type dye-sensitized solar cell based on full Cu_2_O electrodes. J. Alloys Compd..

[37] Zhou W., Yang H., Fang Z. (2007). A novel model for photovoltaic array performance prediction. Appl. Energy.

[38] Frisch M.J., Trucks G.W., Schlegel H.B., Scuseria G.E., Robb M.A., Cheeseman J.R., Scalmani G., Barone V., Mennucci B., Petersson G.A. (2010). Gaussian 09.

[39] Becke A.D. (1993). Density-functional thermochemistry. III. The role of exact exchange. J. Chem. Phys..

[40] Miertuš S., Scrocco E., Tomasi J. (1981). Electrostatic interaction of a solute with a continuum. A direct utilizaion of AB initio molecular potentials for the prevision of solvent effects. Chem. Phys..

[41] Miertuš S., Tomasi J. (1982). Approximate evaluations of the electrostatic free energy and internal energy changes in solution processes. Chem. Phys..

[42] Pascual-Ahuir J.L., Silla E., Tuñón I. (1994). GEPOL: An improved description of molecular surfaces. III. A new algorithm for the computation of a solvent-excluding surface. J. Comp. Chem..

[43] De Proft F., Geerlings P. (2001). Conceptual and Computational DFT in the Study of Aromaticity. Chem. Rev..

[44] Soto-Rojo R., Baldenebro-Lopez J., Glossman-Mitnik D. (2015). Study of chemical reactivity in relation to experimental parameters of efficiency in coumarin derivatives for dye sensitized solar cells using DFT. Phys. Chem. Chem. Phys..

[45] Sun C., Li Y., Song P., Ma F. (2016). An experimental and theoretical investigation of the electronic structures and photoelectrical properties of ethyl red and carminic acid for DSSC application. Materials.

[46] Asbury J.B., Wang Y.-Q., Hao E., Ghosh H.N., Lian T. (2001). Evidences of hot excited state electron injection from sensitizer molecules to TiO_2_ nanocrystalline thin films. Res. Chem. Intermed..

[47] Bentley C.L., Bond A.M., Hollenkamp A.F., Mahon P.J., Zhang J. (2015). Voltammetric Determination of the Iodide/Iodine Formal Potential and Triiodide Stability Constant in Conventional and Ionic Liquid Media. J. Phys. Chem. C.

[48] Zhang J., Kan Y.H., Li H.B., Geng Y., Wu Y., Su Z.M. (2012). How to design proper π-spacer order of the D-π-A dyes for DSSCs? A density functional response. Dyes Pigm..

[49] Megala M., Beulah J., Rajkumar M. (2016). Theoretical study of anthoxanthin dyes for dye sensitized solar cells (DSSCs). J. Comput. Electron..

[50] Qin C., Clark A.E. (2007). DFT characterization of the optical and redox properties of natural pigments relevant to dye-sensitized solar cells. Chem. Phys. Lett..

[51] Chaitanya K., Ju X.-H., Heron B.M. (2014). Theoretical study on the light harvesting efficiency of zinc porphyrin sensitizers for DSSCs. RSC Adv..

[52] Li M., Kou L., Diao L., Zhang Q., Li Z., Wu Q., Lu W., Pan D., Wei Z. (2015). Theoretical study of WS-9-Based organic sensitizers for unusual vis/NIR absorption and highly efficient dye-sensitized solar cells. J. Phys. Chem. C.

[53] Calogero G., Yum J.-H., Sinopoli A., Di Marco G., Grätzel M., Nazeeruddin M.K. (2012). Anthocyanins and betalains as light-harvesting pigments for dye-sensitized solar cells. Sol. Energy.

[54] Tang Y., Gao H., Parrish D.A., Shreeve J.M. (2015). 1,2,4-Triazole Links and N-Azo Bridges Yield Energetic Compounds. Chem. Eur. J..

[55] Zhang Z.L., Zou L.Y., Ren A.M., Liu Y.F., Feng J.K., Sun C.C. (2013). Theoretical studies on the electronic structures and optical properties of star-shaped triazatruxene/heterofluorene co-polymers. Dyes Pigm..

[56] Pounraj P., Mohankumar V., Pandian M.S., Ramasamy P. (2018). The effect of different π-bridge configuration on bi-anchored triphenylamine and phenyl modified triphenylamine based dyes for dye sensitized solar cell (DSSC) application: A theoretical approach. J. Mol. Graph. Model..

[57] Smolyar A.E., Boldyrev A.I., Charkin O.P., Klimenko N.M., Avdeev V.I. (1976). Koopman’s theorem and allowance for the relaxation of MO’s in ab initi calculations of polyhalogenides. J. Struct. Chem..

[58] Manzoor T., Niaz S., Pandith A.H. (2019). Exploring the effect of different coumarin donors on the optical and photovoltaic properties of azo-bridged push-pull systems: A theoretical approach. Int. J. Quantum Chem..

[59] Mohankumar V., Pounraj P., Pandian M.S. (2019). Theoretical Investigation on Flavones and Isoflavones-Added Triphenylamine-Based Sensitizers for DSSC Application. Braz. J. Phys..

[60] Sinopoli A., Calogero G., Bartolotta A. (2019). Computational aspects of anthocyanidins and anthocyanins: A review. Food Chem..

[61] Madugula S.S., Yarasi S. (2017). Molecular design of porphyrin dyes for dye sensitized solar cells: A quantitative structure property relationship study. Int. J. Quantum Chem..

[62] Hajizadeh F., Reisi-Vanani A., Azar Y.T. (2018). Theoretical design of Zn-dithiaporphyrins as sensitizer for dye-sensitized solar cells. Curr. Appl. Phys..

[63] Lu L., Yu L. (2014). Understanding Low Bandgap Polymer PTB7 and Optimizing Polymer Solar Cells Based on It. Adv. Mater..

[64] Kranthiraja K., Kim S., Lee C., Gunasekar K., Sree V.G., Gautam B., Gundogdu K., Jin S.-H., Kim B.J. (2017). The Impact of Sequential Fluorination of Conjugated Polymers on Charge Generation in All-Polymer Solar Cells. Adv. Funct. Mater..

[65] Terenti N., Giurgi G.-I., Szolga L., Stroia I., Terec A., Grosu I., Crisan A.P. (2022). Effect of the Terminal Acceptor Unit on the Performance of Non-Fullerene Indacenodithiophene Acceptors in Organic Solar Cells. Molecules.

