# Supplementary material for: Experimental and Computational Studies on Bio-Inspired Flavylium Salts as Sensitizers for Dye-Sensitized Solar Cells
Source: Materials (Basel). 2022 Oct 8;15(19):6985. doi: 10.3390/ma15196985 (PMC9572272; doi:10.3390/ma15196985)
Supplement: Supplementary file 1 [file materials-15-06985-s001.zip › materials-1919704-supplementary.pdf]

*Supplementary Material*

# Experimental and Computational Studies on Bio-Inspired Flavylium Salts as Sensitizers for Dye-Sensitized Solar Cells

**Iulia Păușescu [1], Anamaria Todea [1], Diana-Maria Dreavă [1], Tania Boboescu [1], Bianca Pațcan [1], Larisa Pațcan [1], Daiana Albulescu [1,2], Valentin Badea [1], Francisc Peter [1], Róbert Tőtős [3], Daniel Ursu [2], Lorant Szolga [4,*] and Mihai Medeleanu [1,*]**

[1] Faculty of Industrial Chemistry and Environmental Engineering, Politehnica University of Timişoara, Carol Telbisz 6, 300001 Timisoara, Romania

[2] National Institute of Research and Development for Electrochemistry and Condensed Matter, Dr A. Păunescu Podeanu 144, 300569 Timisoara, Romania

[3] Faculty of Chemistry and Chemical Engineering, Babes Bolyai University, Arany Janos 11, 400028 Cluj-Napoca, Romania

[4] Optoelectronics Group, Base of Electronics Department, ETTI, Technical University of Cluj-Napoca, 28 Memorandumului Str., 400114 Cluj-Napoca, Romania

* Correspondence: lorant.szolga@bel.utcluj.ro (L.S.); mihai.medeleanu@upt.ro (M.M.)

*NMR spectra*

**NMR** spectra were recorded on a Bruker AVANCE III spectrometer (Bruker Daltonik GmbH, Bremen, Germany) operating at 500.0 MHz ($^1$H) and 125.0 MHz ($^{13}$C) at 298 K. Chemical shifts δ are reported in ppm versus tetramethylsilane, TMS, coupling constants are reported in Hz, and the following abbreviations are used for splitting pattern: s (singlet), d (doublet), dd (doublet of doublets) and m (multiplet). The samples were dissolved in DMSO-*d*$_6$.

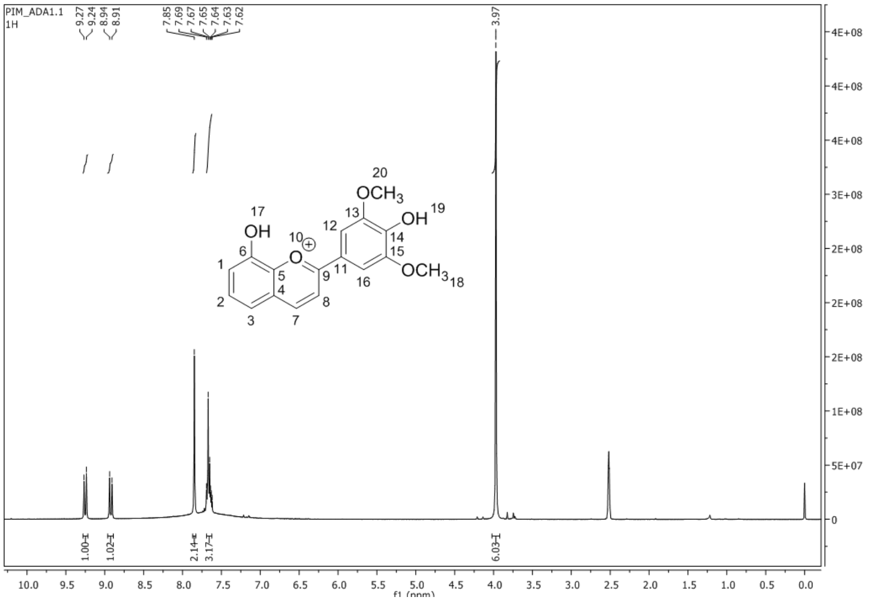


Figure S1. $^1$H-NMR spectrum of compound **1**

**$^1$H-NMR** (500 MHz, DMSO-*d*$_6$, δ ppm): 3.97 (s, 6H, **H18**, **H20**); 7.62-7.64 (m, 3H, **H1**, **H2**, **H3**); 7.85 (s, 2H, **H12**, **H16**); 8.91 (d, 1H, *J*=9.24 Hz, **H8**); 9.25 (d, 1H, *J*=9.24 Hz**, H7**).

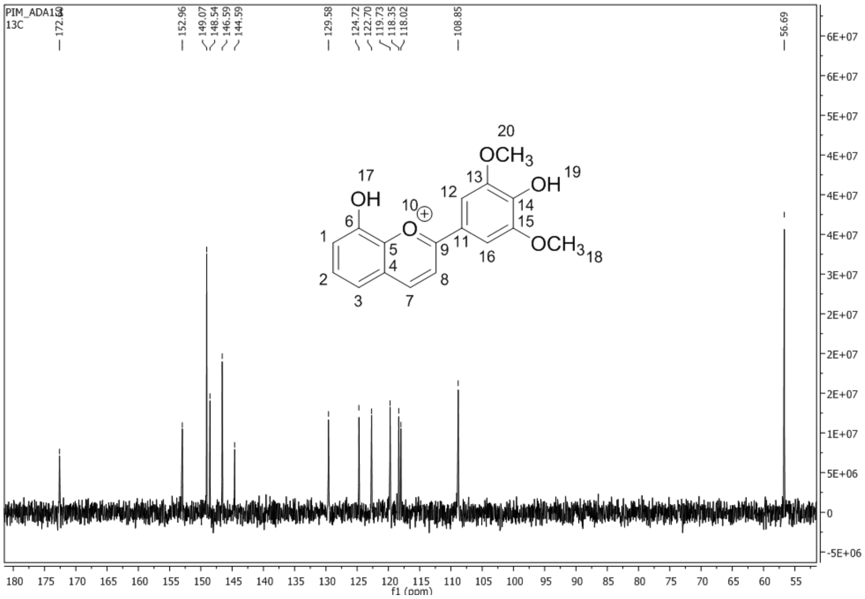


Figure S2. $^{13}$C-NMR spectrum of compound **1**

**$^{13}$C-NMR** (125 MHz, DMSO-*d*$_6$, δ ppm): 56.6 (**C18, C20**); 108.8 (**C12, C16**); 118.0 (**C8**); 118.3 (**C4**); 119.7 (**C1**); 122.7 (**C3**); 124.7 (**C4**); 129.5 (**C2**); 144.5 (**C6**); 146.5 (**C5**); 148.5 (**C13, C15**); 149.0 (**C14**); 152.9 (**C7**); 172.6 (**C9**).

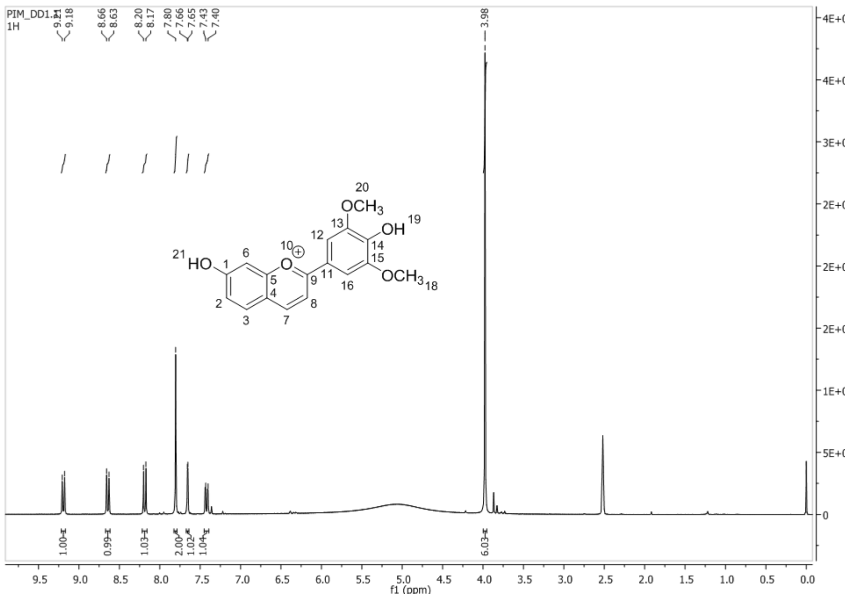

Figure S3. $^{1}$H-NMR spectrum of compound **2**

**$^{1}$H-NMR** (500 MHz, DMSO-*d$_6$*, δ ppm): 3.98 (s, 6H**, H18, H20**); 7.41 (dd, 1H, *J*=8.95, 2.2 Hz, **H2**); 7.66 (d, 1H, *J*=2.2 Hz, **H6**); 7.80 (s, 2H, **H12, H16**); 8.19 (d, 1H, *J*=8.95 Hz, **H3**); 8.65 (d, 1H, *J*=8.85 Hz, **H8**); 9.20 (d, 1H, *J*=8.85 Hz, **H7**).

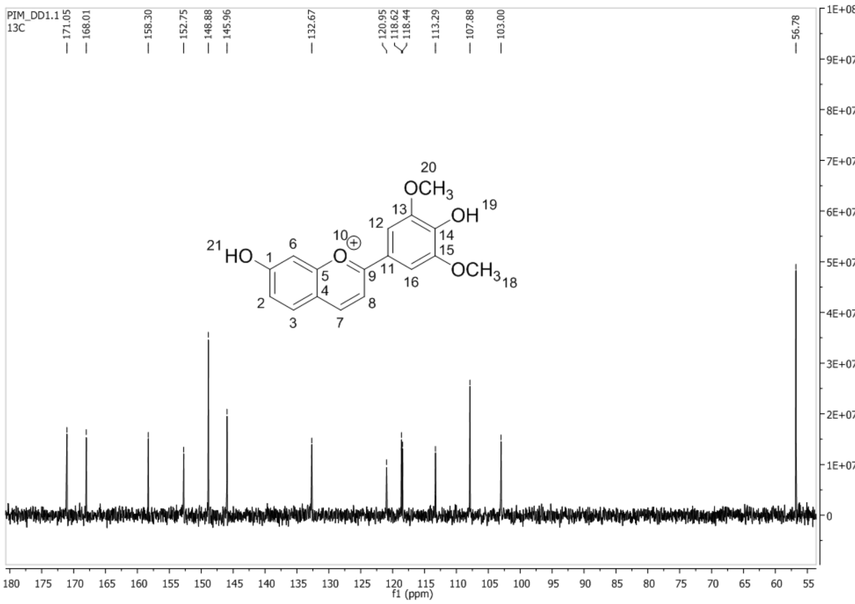

Figure S4. $^{13}$C-NMR spectrum of compound **2**

**$^{13}$C-NMR** (125 MHz, DMSO-*d$_6$*, δ ppm): 56.7 (**C18, C20**); 103.0 (**C6**); 107.8 (**C12, C16**); 113.2 (**C8**); 118.4 (**C11**); 118.6 (**C4**); 120.9 (**C2**); 132.6 (**C3**); 145.9 (**C14**); 148.8 (**C13, C15**); 152.7 (**C7**); 158.3 (**C5**); 168.0 (**C1**); 171.0 (**C9**).

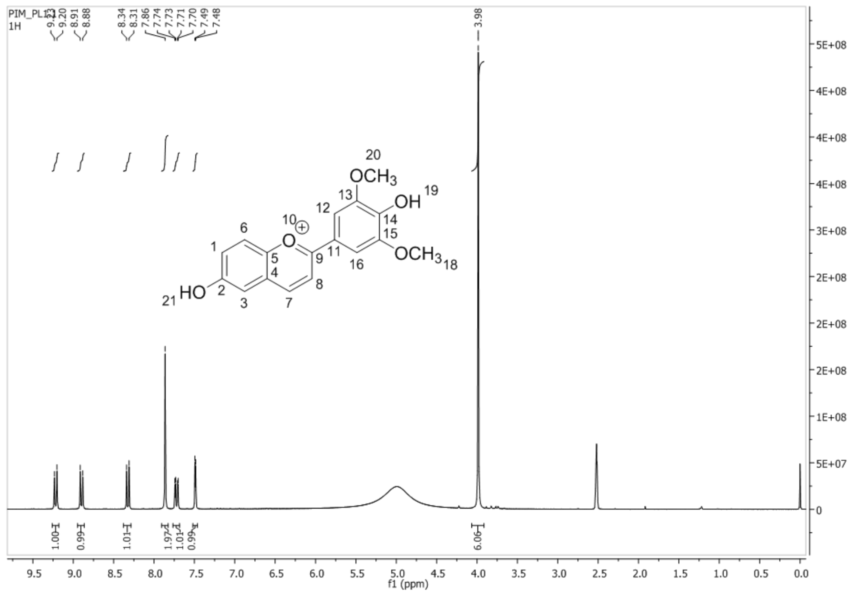


Figure S5. $^{1}$H-NMR spectrum of compound **3**

**$^{1}$H-NMR** (500 MHz, DMSO-$d_6$, δ ppm): 3.98 (s, 6H, **H18, H20**); 7.49 (d, 1H, *J*=2.90 Hz, **H3**); 7.72 (dd, 1H, *J*=9.29, 2.90 Hz, **H1**); 7.86 (s, 2H, **H12, H16**); 8.33 (d, 1H, *J*=9.30 Hz, **H6**); 8.90 (d, 1H, *J*=9.32 Hz, **H8**); 9.21 (d, 1H, *J*=9.32 Hz, **H7**).

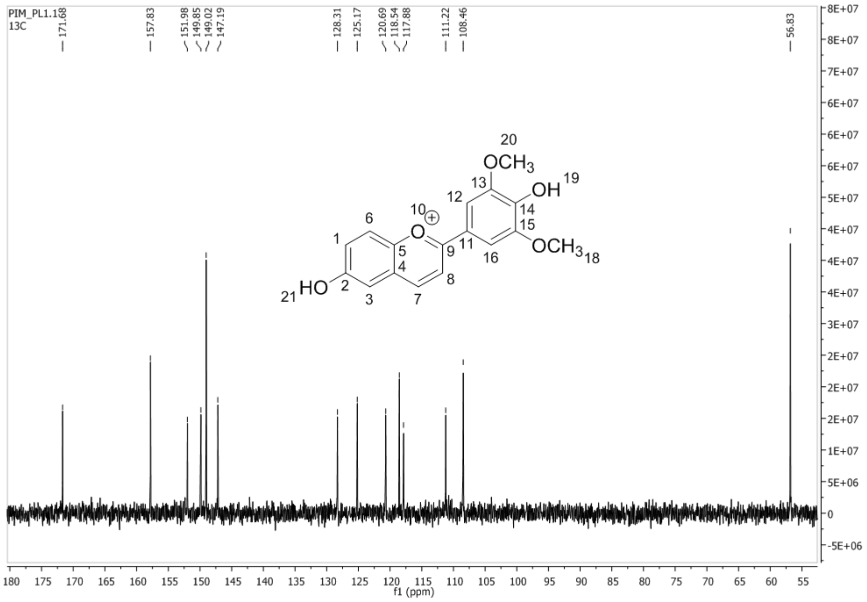


Figure S6. $^{13}$C-NMR spectrum of compound **3**

**$^{13}$C-NMR** (125 MHz, DMSO-$d_6$, δ ppm): 56.8 (**C18, C20**); 108.4 (**C12, C16**); 111.2 (**C3**); 117.8 (**C8**); 118.5 (**C14**); 120.6 (**C6**); 125.1 (**C4**); 128.3 (**C1**); 147.1 (**C11**); 149.0 (**C13, C15**); 149.8 (**C5**); 151.9 (**C7**); 157.8 (**C2**); 171.6 (**C9**).

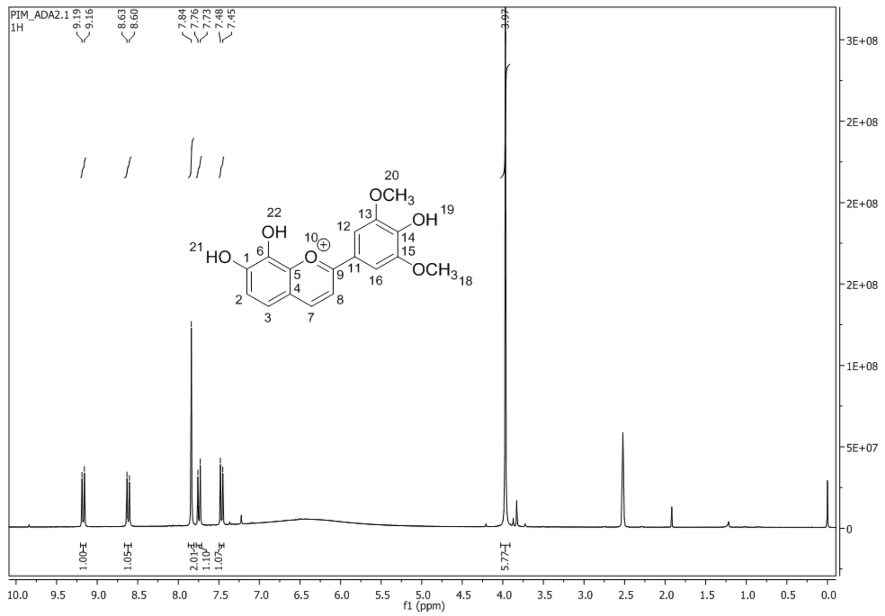

Figure S7. $^{1}$H-NMR spectrum of compound **4**

**$^{1}$H-NMR** (500 MHz, DMSO-*d$_6$*, δ ppm): 3.97 (s, 6H, **H18, H20**); 7.46 (d, 1H, *J*=8.84 Hz, **H2**); 7.74 (d, 1H, *J*=8.84 Hz, **H3**); 7.84 (s, 2H, **H12, H16**); 8.62 (d, 1H, *J*=8.90 Hz, **H8**); 9.17 (d, 1H, *J*=8.90 Hz, **H7**).

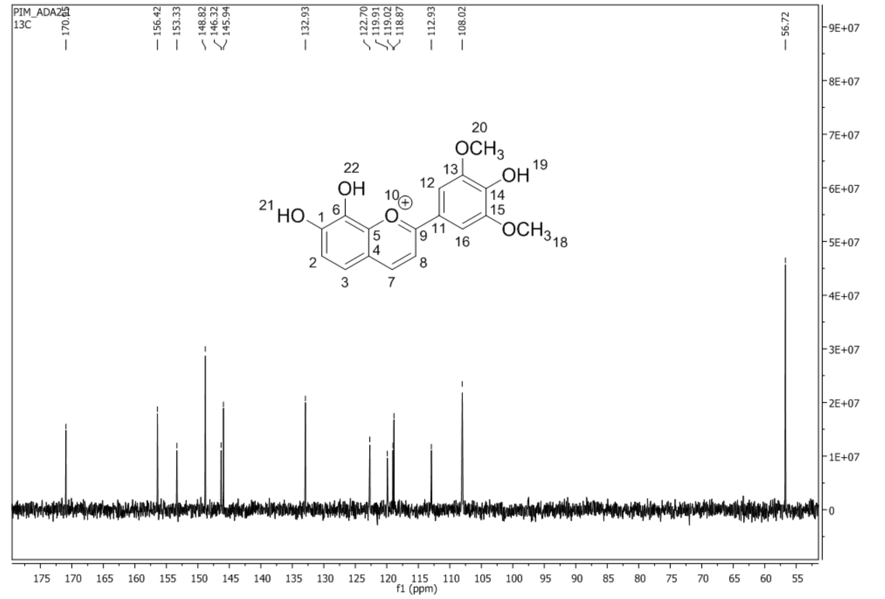

Figure S8. $^{13}$C-NMR spectrum of compound **4**

**$^{13}$C-NMR** (125 MHz, DMSO-*d$_6$*, δ ppm): 56.7 (**C18, C20**); 108.0 (**C12, C16**); 112.9 (**C8**); 118.8 (**C5**); 119.0 (**C4**); 119.9 (**C2**); 122.7 (**C3**); 132.9 (**C6**); 145.9 (**C11**); 146.3 (**C14**); 148.8 (**C13, C15**); 153.3 (**C7**); 156.4 (**C1**); 170.9 (**C9**).

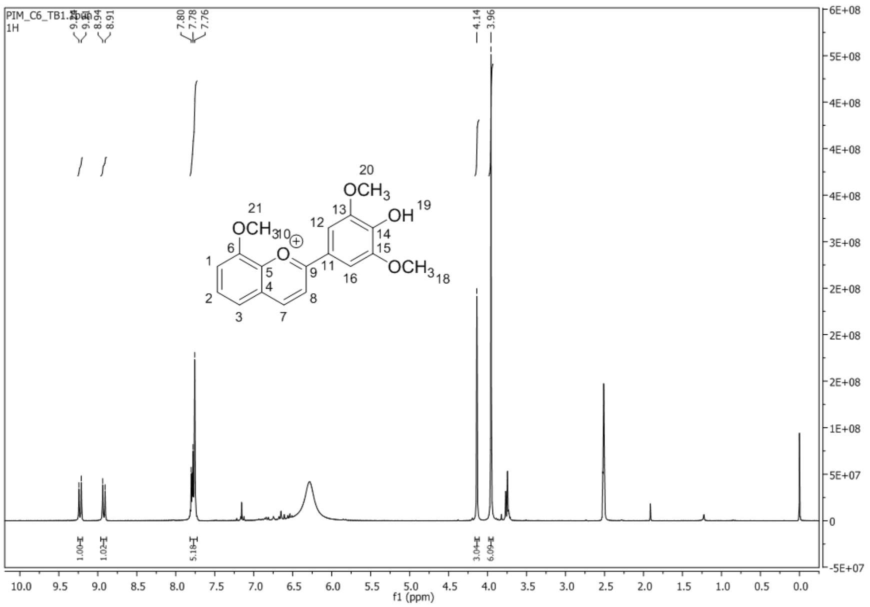


Figure S9. $^{1}$H-NMR spectrum of compound **5**

**$^{1}$H-NMR** (500 MHz, DMSO-*d*$_6$, δ ppm): 3.96 (s, 6H, **H18, H20**); 4.14 (s, 3H, **H21**); 7.80-7.76 (m, 5H, **H1, H2, H3, H12, H16**); 8.93 (d, 1H, *J*=9.33 Hz, **H8**); 9.22 (d, 1H, *J*=9.33 Hz, **H7**).

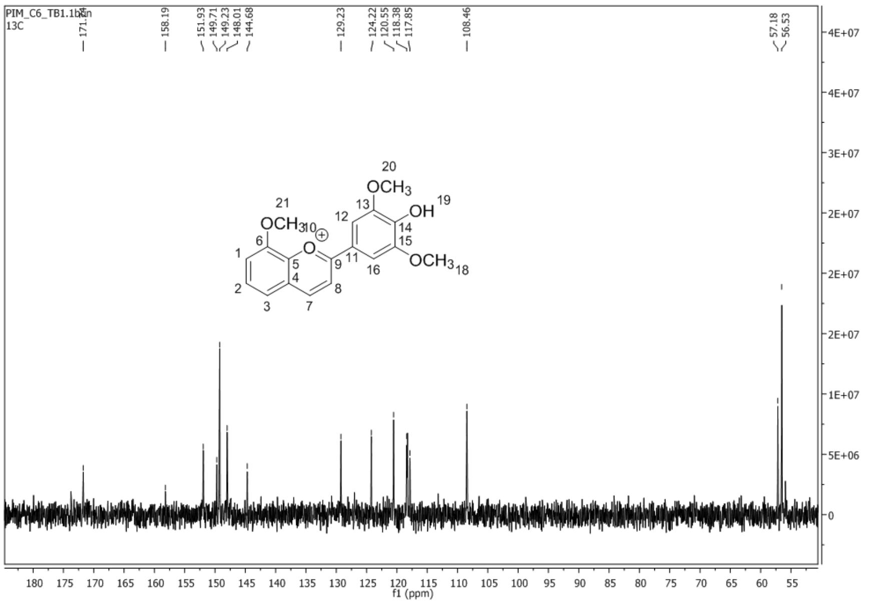


Figure S10. $^{13}$C-NMR spectrum of compound **5**

**$^{13}$C-NMR** (125 MHz, DMSO-*d*$_6$, δ ppm): 56.5 (**C18, C20**); 57.1 (**C21**); 108.4 (**C12, C16**); 117.8 (**C8**); 118.3 (**C1**); 120.5 (**C3**); 124.2 (**C11**); 129.2 (**C2**); 144.6 (**C4**); 148.0 (**C14**); 149.2 (**C13, C15**); 149.7 (**C5**); 151.9 (**C7**); 158.1 (**C6**); 171.7 (**C9**).

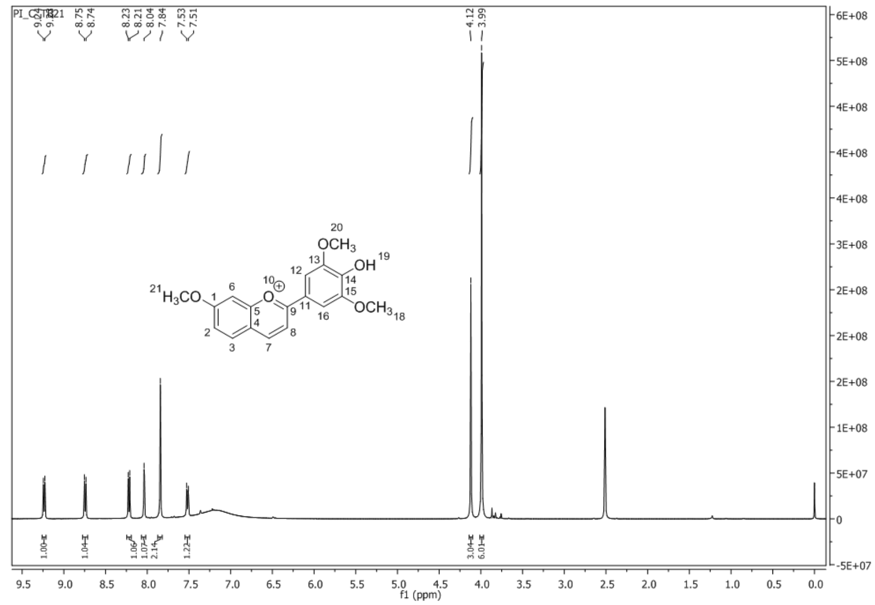


Figure S11. $^{1}$H-NMR spectrum of compound **6**

**$^{1}$H-NMR** (500 MHz, DMSO-*d*$_6$, δ ppm): 3.99 (s, 6H, **H18, H20**); 4.12 (s, 3H, **H21**); 7.52 (d, 1H, *J*=10.6 Hz, **H2**); 7.84 (s, 2H, **H12, H16**); 8.04 (s, 1H, **H6**); 8.22 (d, 1H, *J*=9.0 Hz, **H3**); 8.75 (d, 1H, *J*=8.9 Hz, **H8**); 9.24 (d, 1H, *J*=8.9 Hz, **H7**).

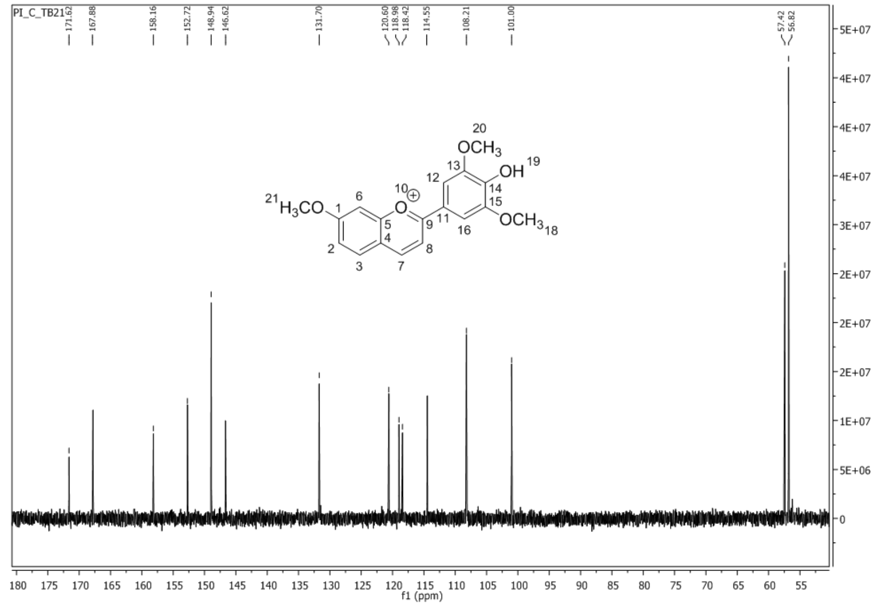


Figure S12. $^{13}$C-NMR spectrum of compound **6**

**$^{13}$C-NMR** (125 MHz, DMSO-*d*$_6$, δ ppm): 56.8 (**C18, C20**); 57.4 (**C21**); 101.0 (**C6**); 108.2 (**C12, C16**); 114.5 (**C8**); 118.4 (**C11**); 118.9 (**C4**); 120.6 (**C2**); 131.7 (**C3**); 146.6 (**C14**); 148.9 (**C13, C15**); 152.7 (**C7**); 158.1 **(C5**); 167.8 (**C1**); 171.6 (**C9**).

Table S1. The main characterization parameters of the synthesized flavylium dyes

| Compound | NMR purity (%) | LC-MS ([M]+, amu) | λ (nm) | m.p. (°C) |
|---|---|---|---|---|
| **1** | 96 | 299 | 470 | 224-227 |
| **2** | 95 | 299 | 482 | 226-229 |
| **3** | 98 | 299 | 490 | 207-210 |
| **4** | 97 | 315 | 496 | 187-190 |
| **5** | 98 | 313 | 478 | 234-237 |
| **6** | 98 | 313 | 481 | 224-227 |

*LC-MS data*

**LC-MS** measurements were performed on an Agilent 1200 HPLC system coupled with Agilent 6410B triple Quadrupole Mass Spectrometer (Agilent Technologies, USA), which was equipped with an electrospray ion source. The samples were dissolved in methanol, the separation was performed by isocratic elution using acetonitrile 40%/$H_2O$ and 60% formic acid 0.1% at a flow rate of 0.3 mL/min. The runtime was 7 min. The mass spectrometer was operated in positive ionization mode. For all measurements the ion source was set to 350 ºC and the capillary voltage was set to 4kV. The results are given as [M]+, not as [M+H]+ as it is commonly when using ESI, because the compounds are already in ionic form.

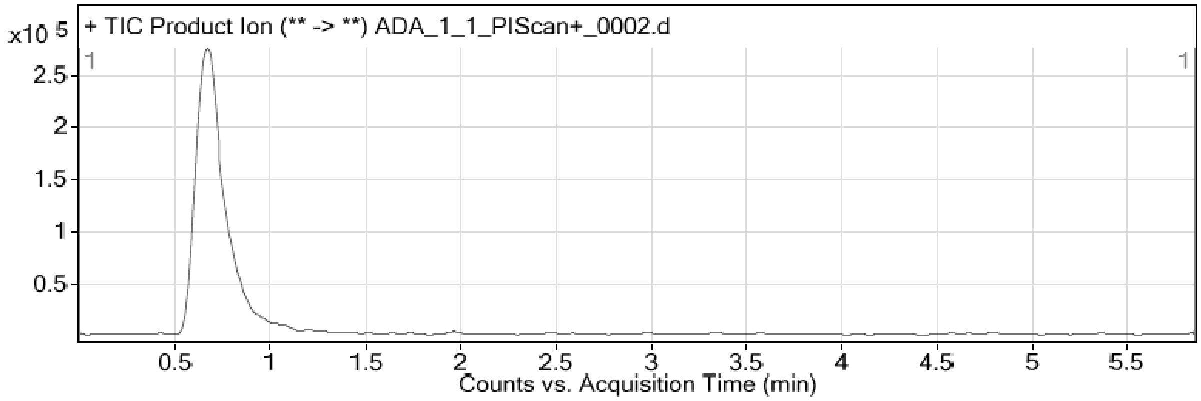


Figure S13. Chromatogram of dye **1** (fragmentor voltage 120V, collision energy 25)

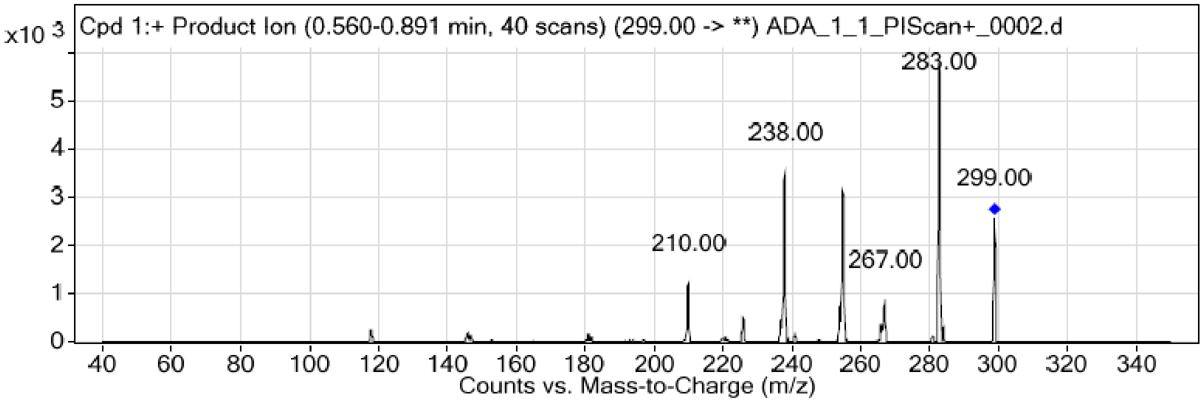


Figure S14. Mass spectrum of dye **1**

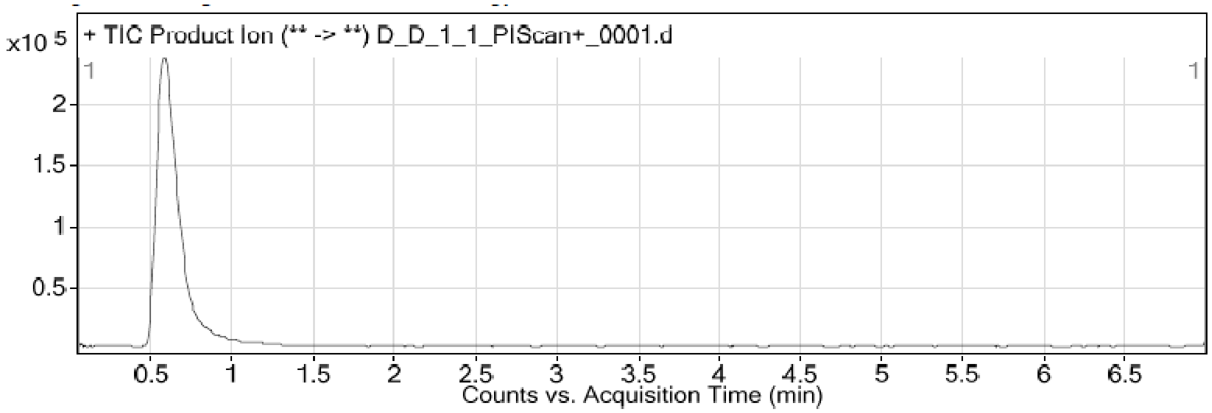


Figure S15. Chromatogram of dye **2** (fragmentor voltage 120V, collision energy 20)

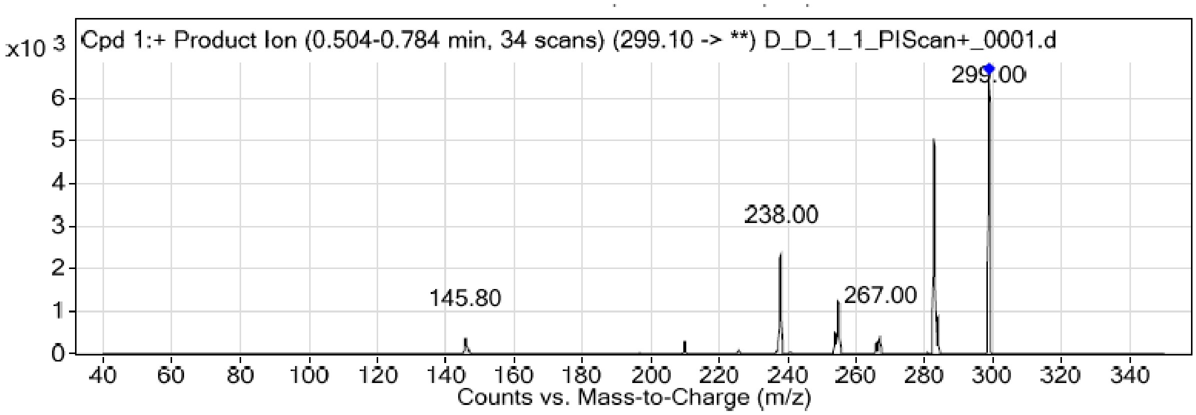


Figure S16. Mass spectrum of dye **2**

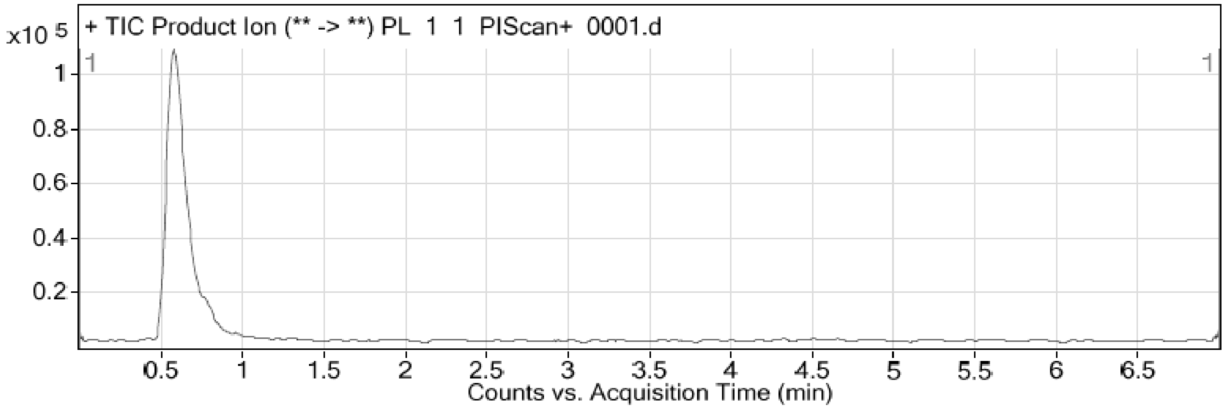


Figure S17. Chromatogram of dye **3** (fragmentor voltage 120V, collision energy 20)

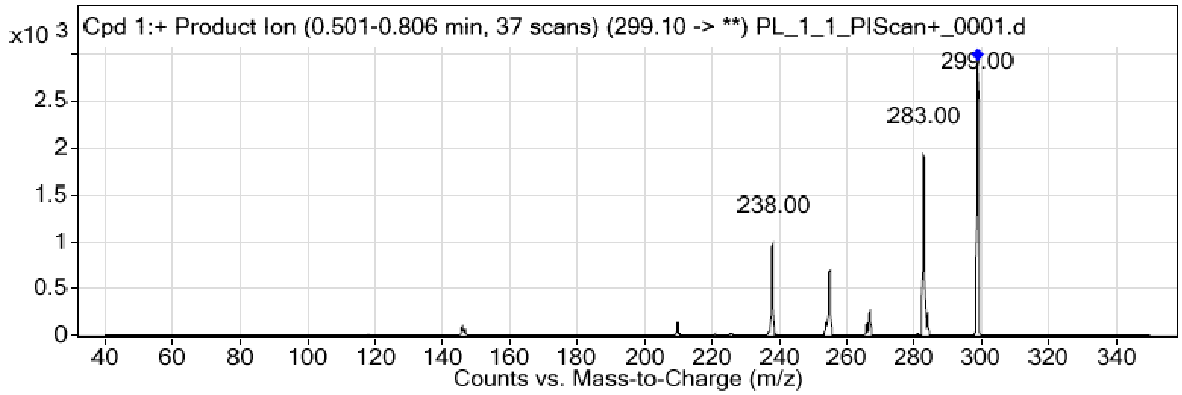


Figure S18. Mass spectrum of dye **3**

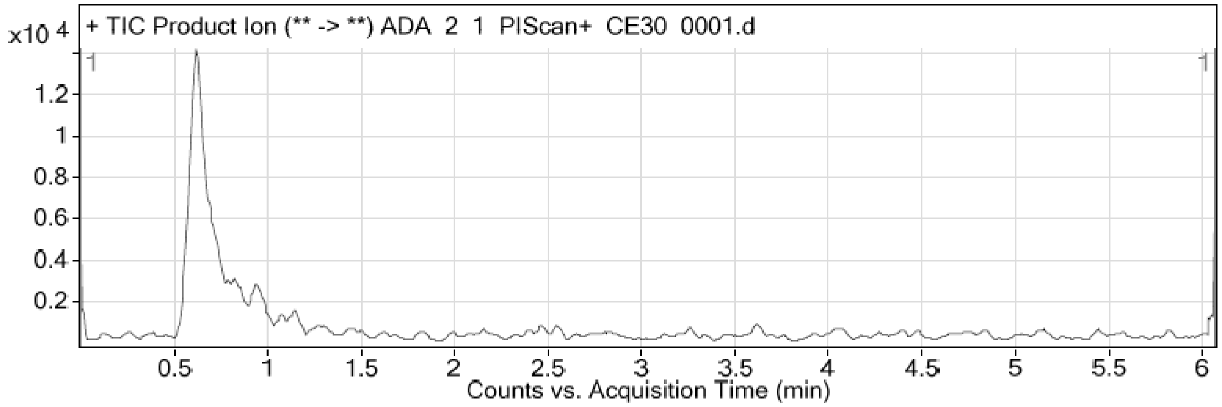


Figure S19. Chromatogram of dye **4** (fragmentor voltage 90V, collision energy 30)

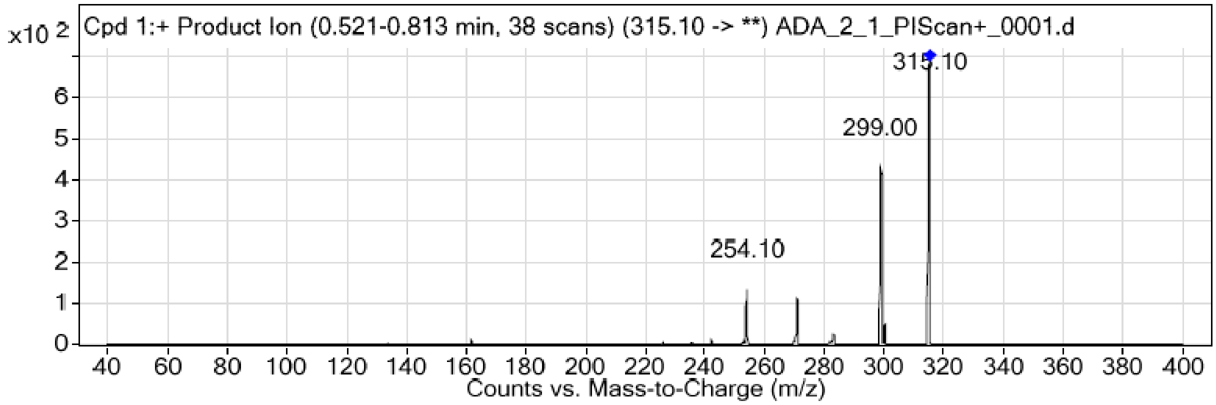


Figure S20. Mass spectrum of dye **4**

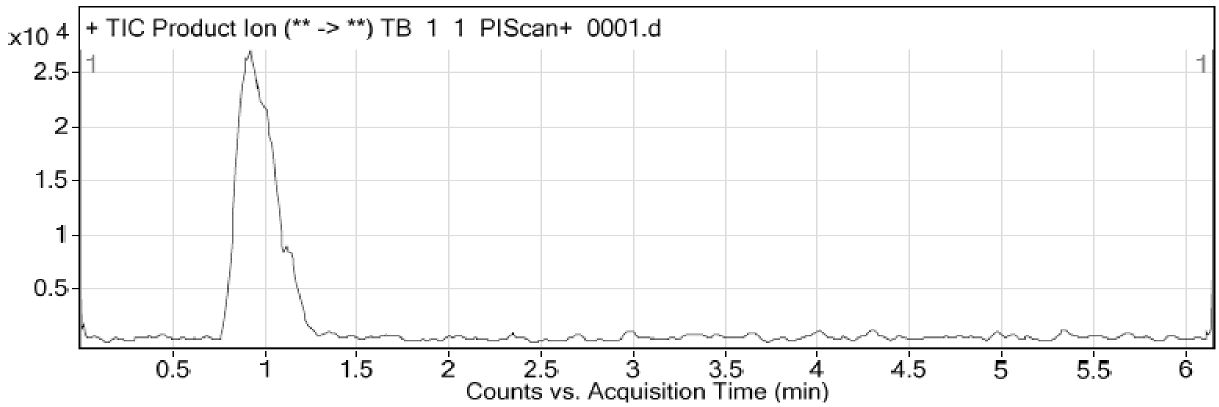


Figure S21. Chromatogram of dye **5** (fragmentor voltage 120V, collision energy 20)

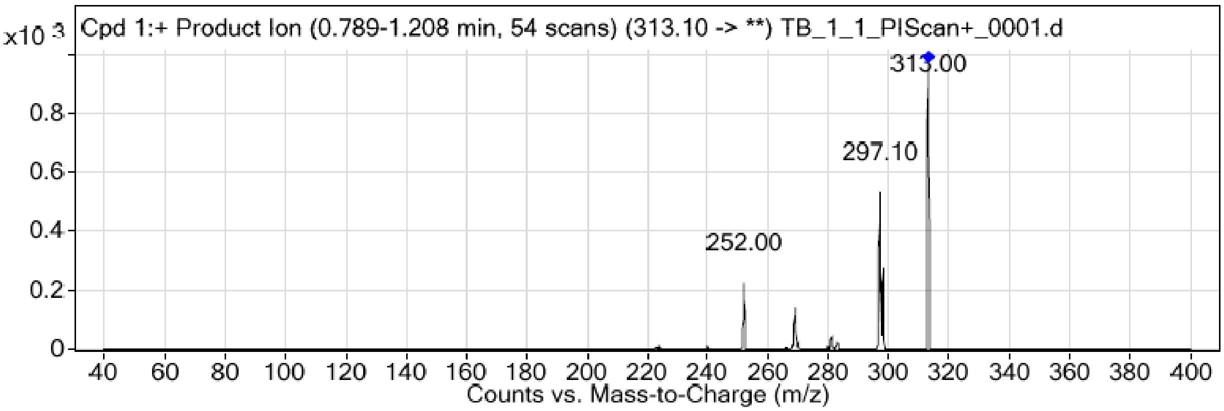


Figure S22. Mass spectrum of dye **5**

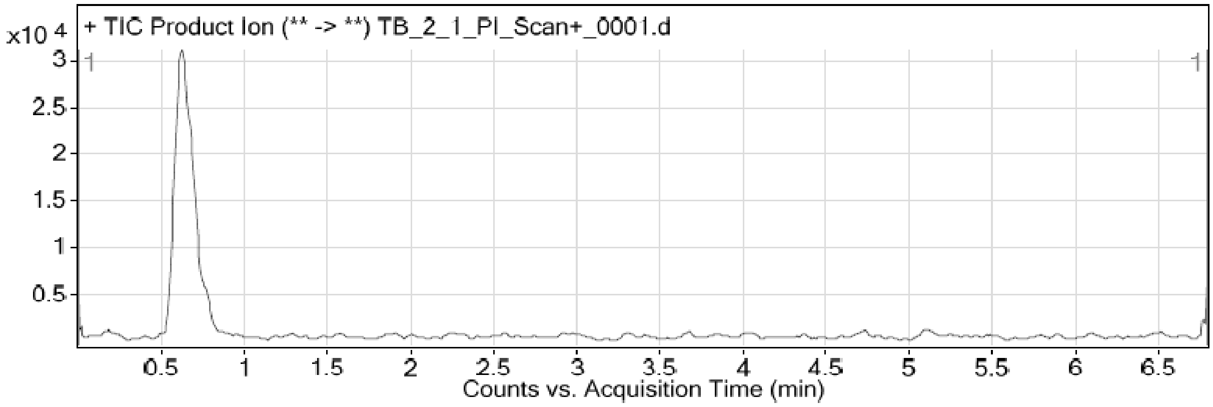


Figure S23. Chromatogram of dye **6** (fragmentor voltage 120V, collision energy 30)

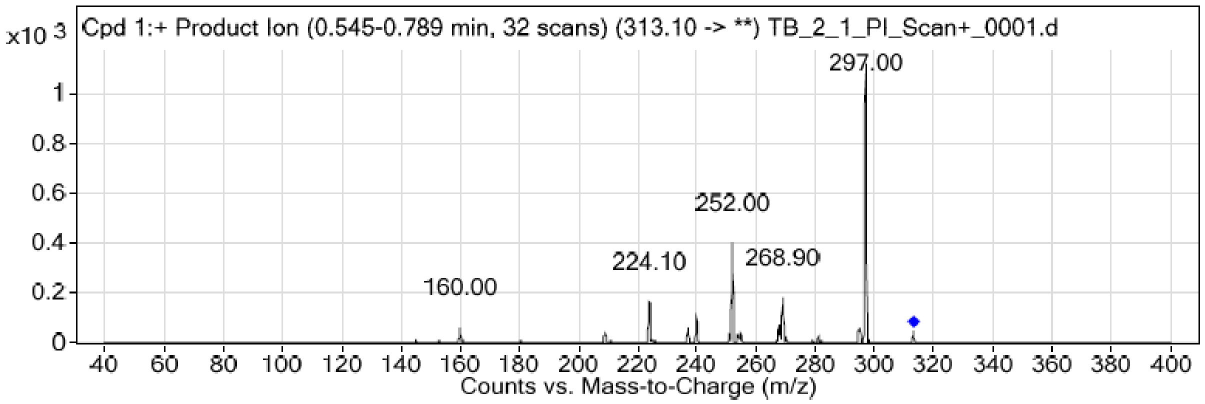


Figure S24. Mass spectrum of dye **6**

### *UV-Vis spectra*

The pH-dependent photochromic properties of the synthesized dye were evaluated through an UV-VIS spectroscopy study. UV-VIS spectra of dye solutions at pH ranging from 2 to 12 were registered over time. Upon pH shifts, color changes were observed, and the overlaid collected UV-Vis spectra for the synthesized dyes are presented in Figures S25 to S35. This proved the existence of multiple species at different pH values.

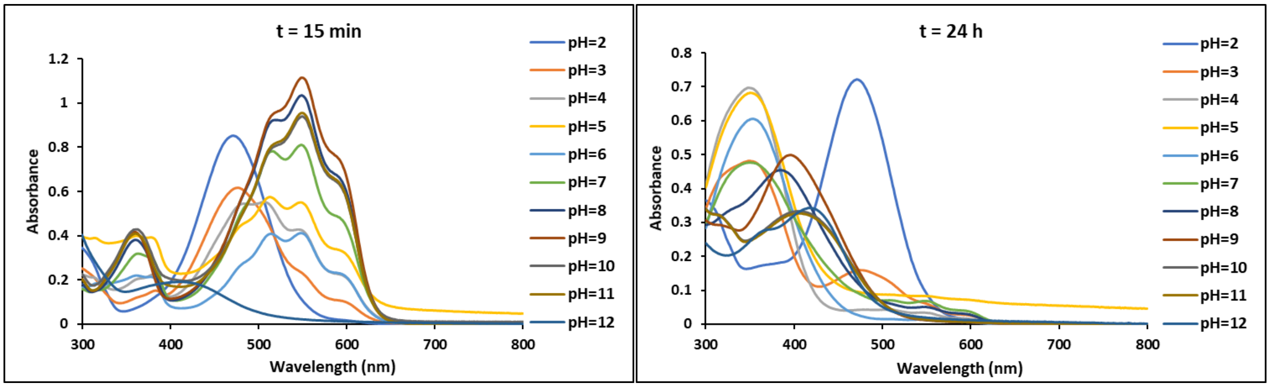


Figure S25. UV-Vis spectra of **1** in different pH buffer solutions

($5{\cdot}10^{-5}$ M in methanol:water 1:14)

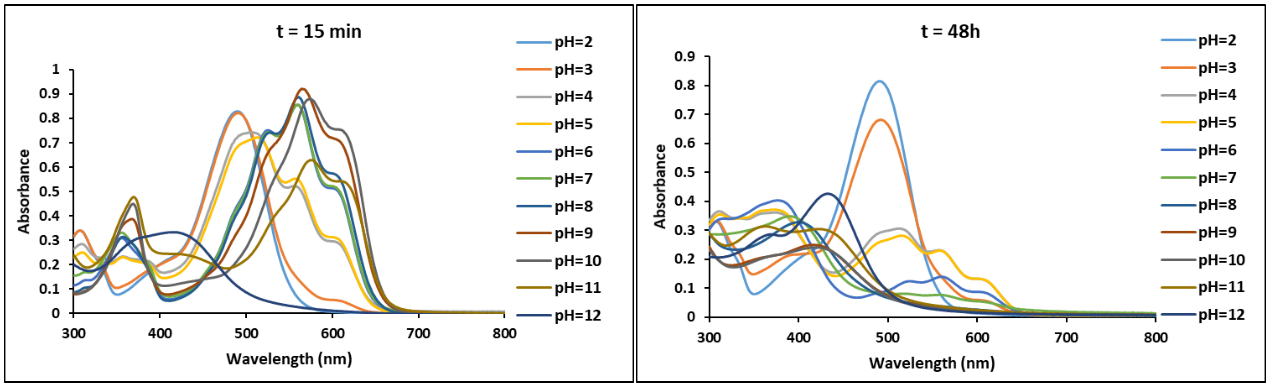


Figure S26. UV-Vis spectra of **3** in different pH buffer solutions

(1.2·$10^{-4}$ M in methanol:water 1:5)

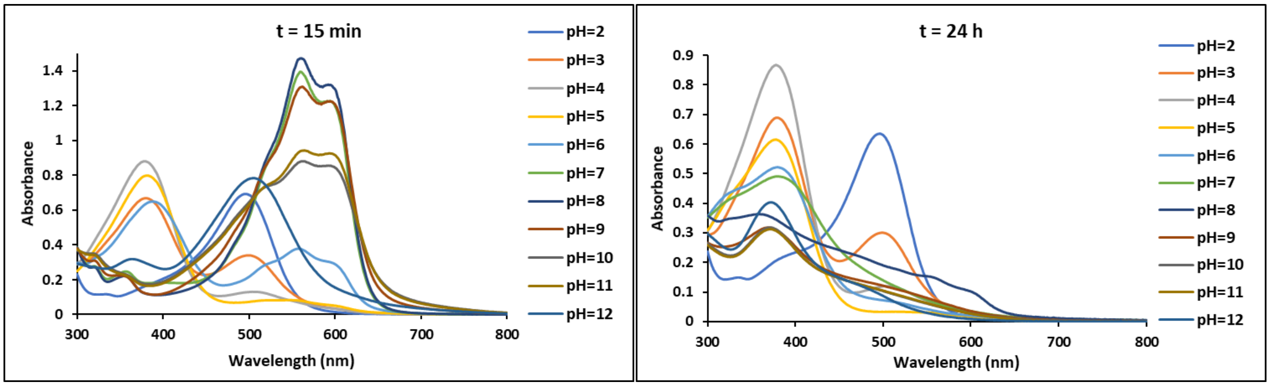


Figure S27. UV-Vis spectra of **4** in different pH buffer solutions

(4.8·$10^{-5}$ M in methanol:water 1:14)

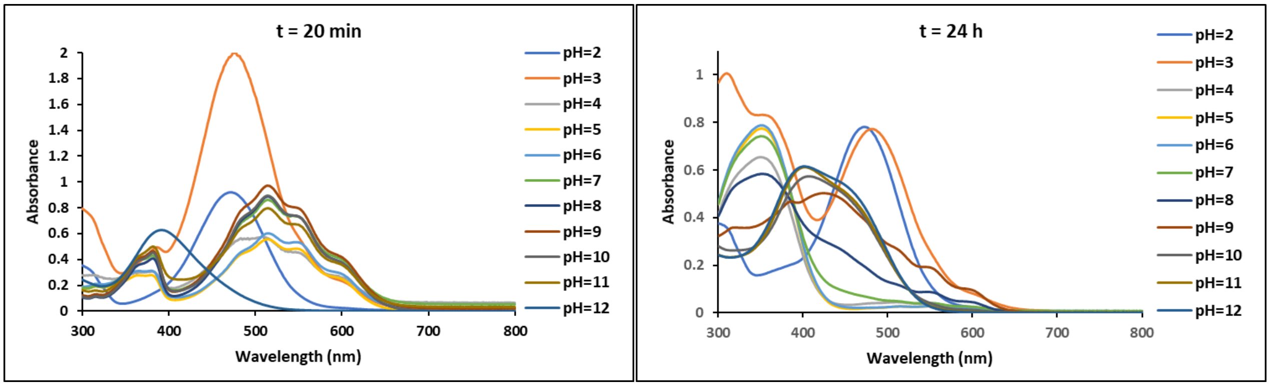


Figure S28. UV-Vis spectra of **5** in different pH buffer solutions

(3.2·$10^{-5}$ M in methanol:water 1:19)

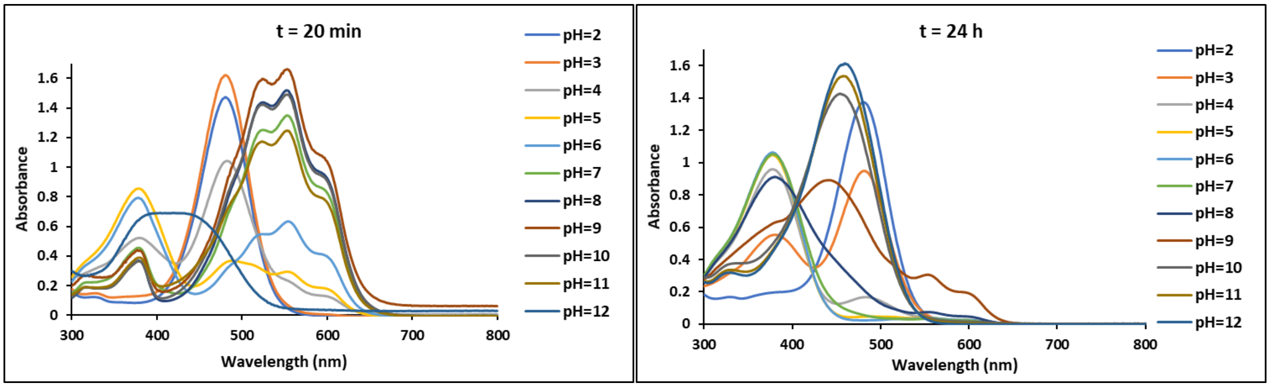


Figure S29. UV-Vis spectra of **6** in different pH buffer solutions (3.2·$10^{-5}$ M in methanol:water 1:19)

Based on the UV-vis spectra collected in time for each pH and dye (Figures S30-S35) several species from the network of chemical reactions responsible for the halochromic behavior were identified and the stability of the species at different pH values was evaluated.

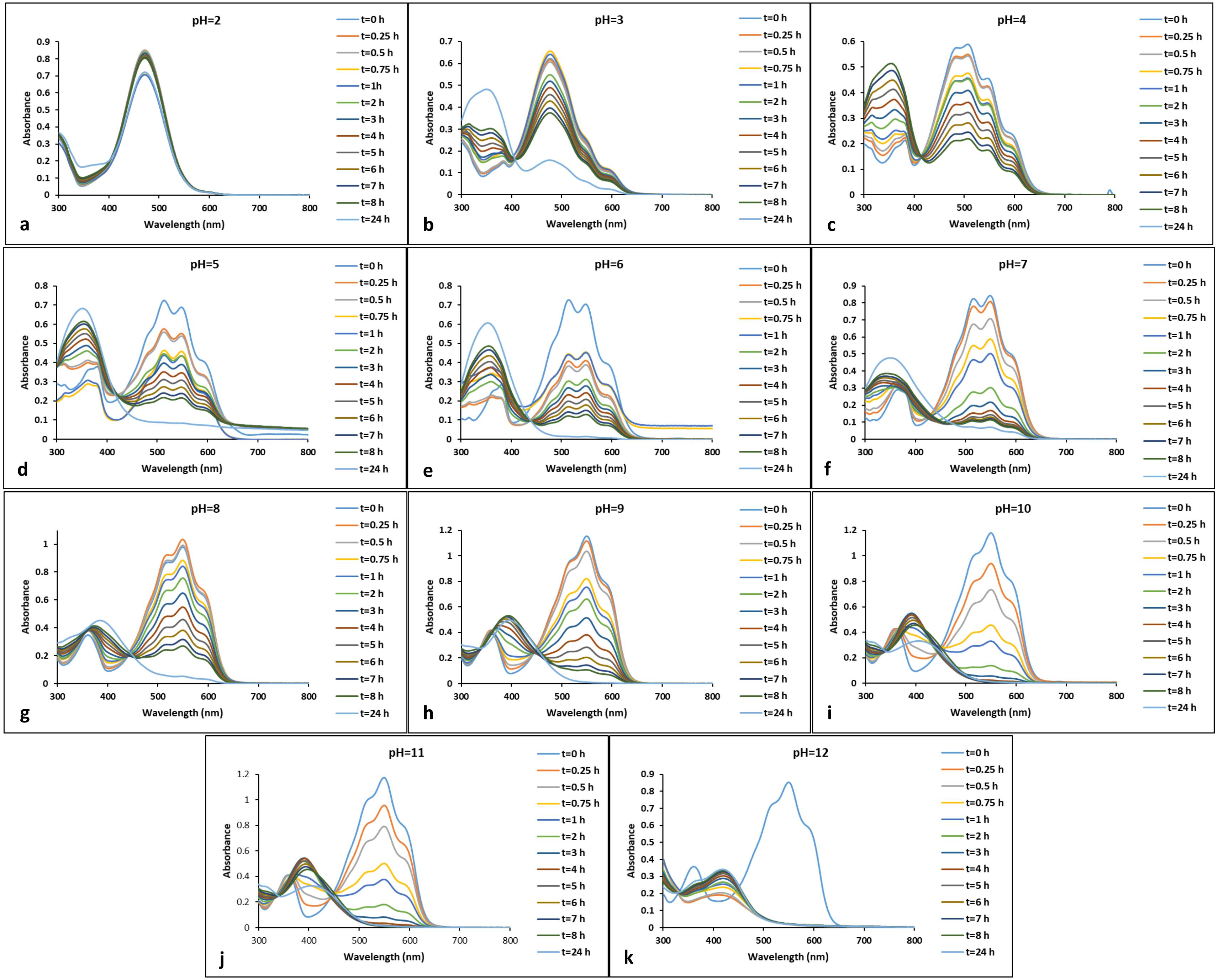


Figure S30. UV-Vis spectra of **1** at pH values from 2 to 12 in time

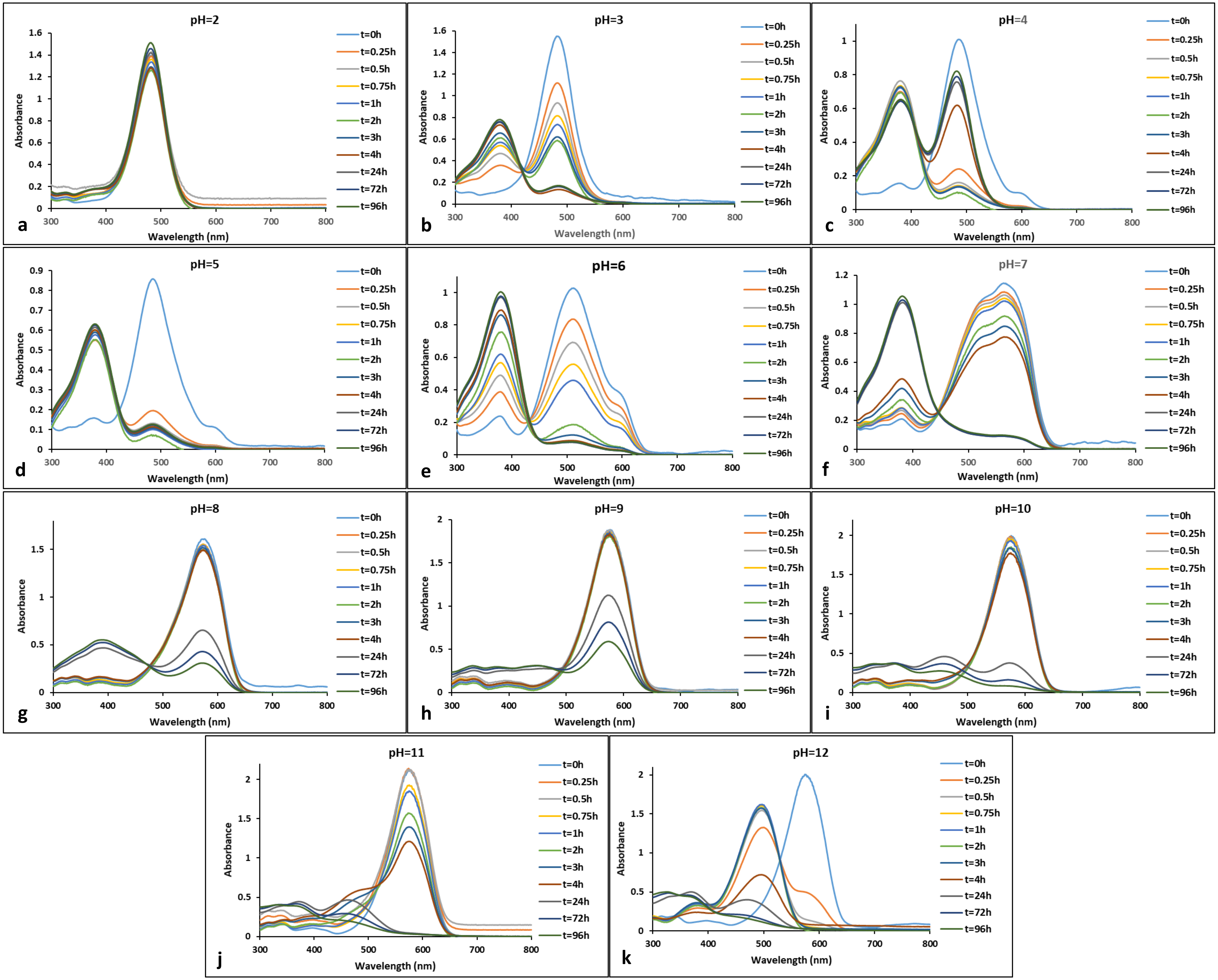


Figure S31. UV-Vis spectra of **2**

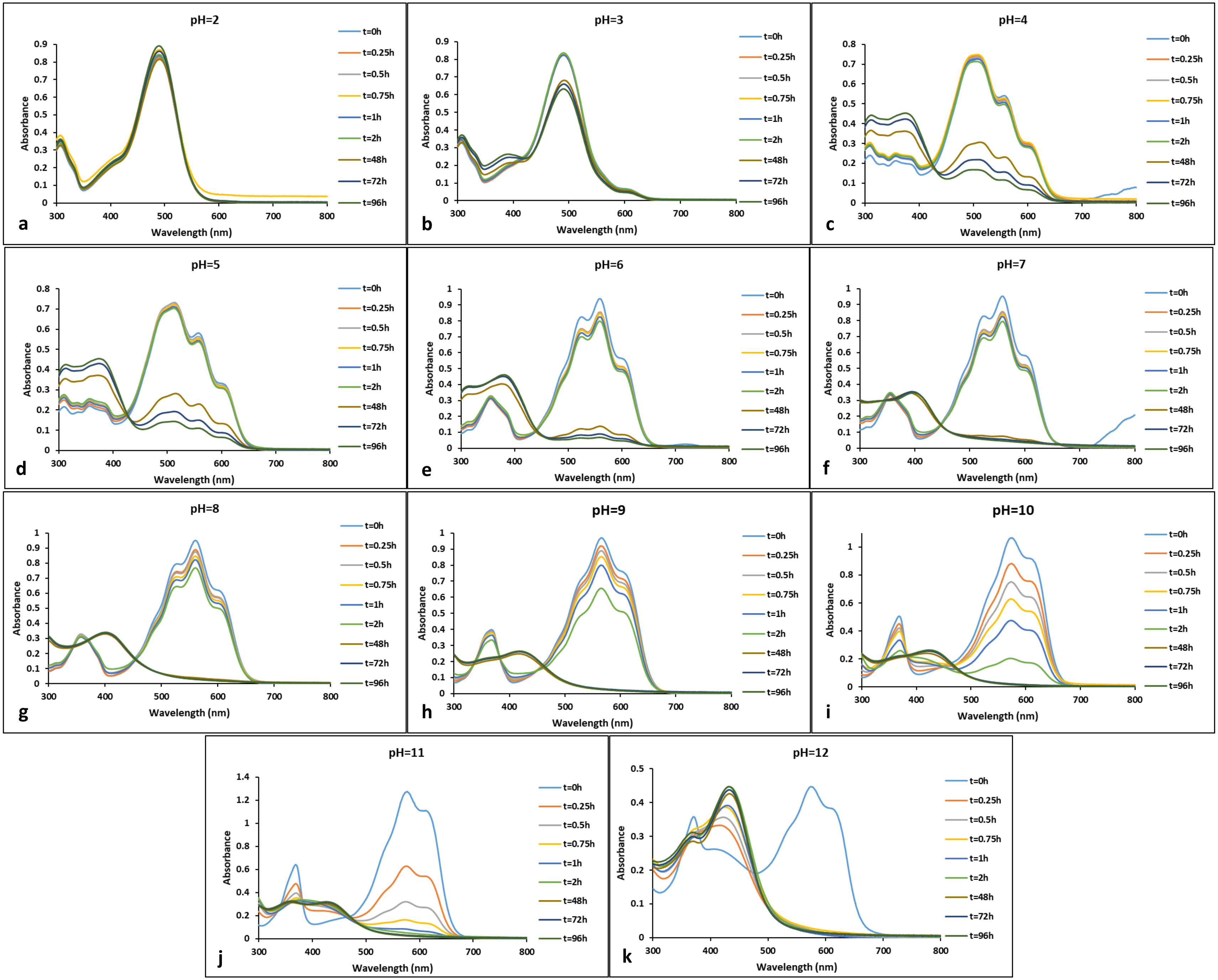


Figure S32. UV-Vis spectra of **3**

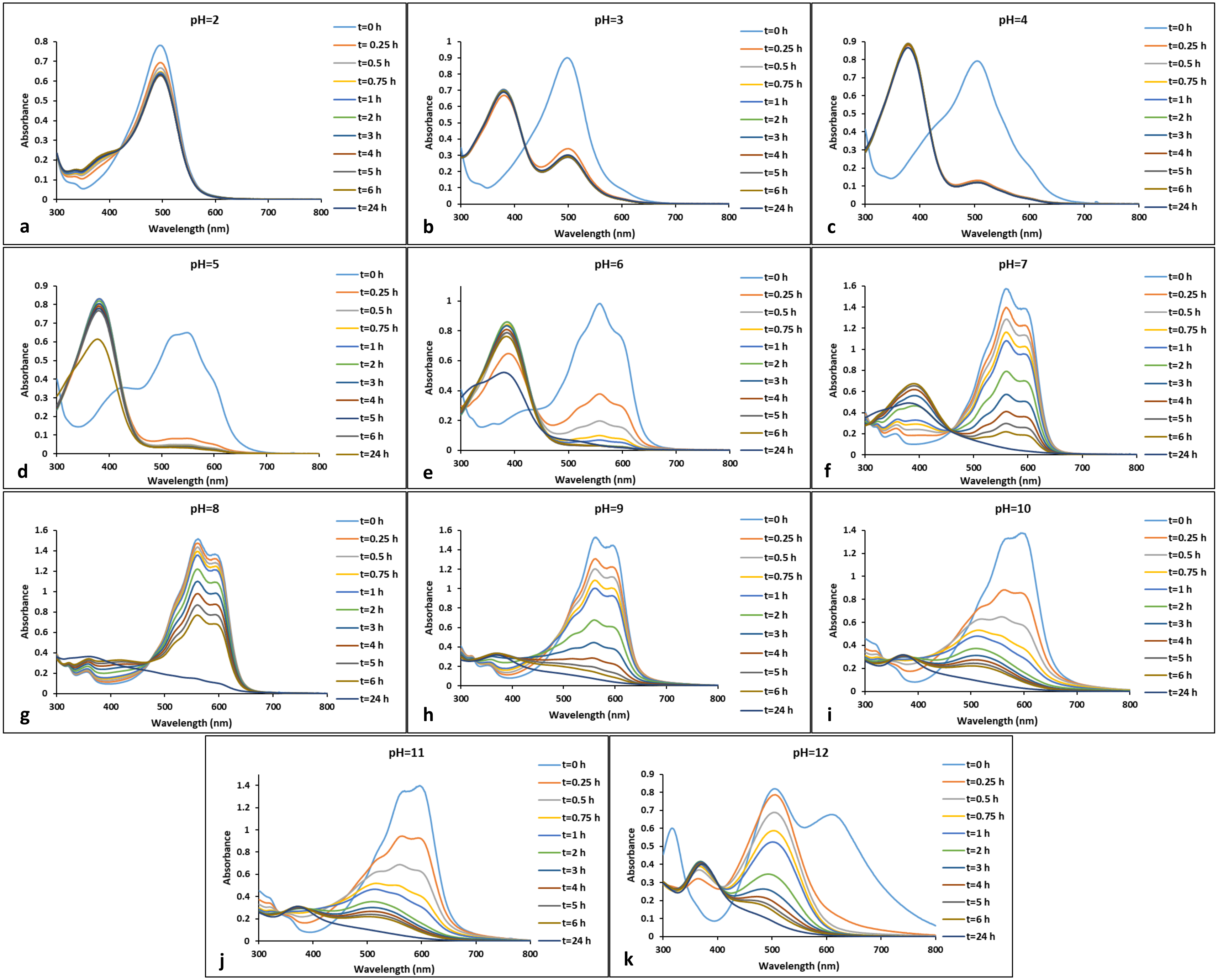


Figure S33. UV-Vis spectra of **4**

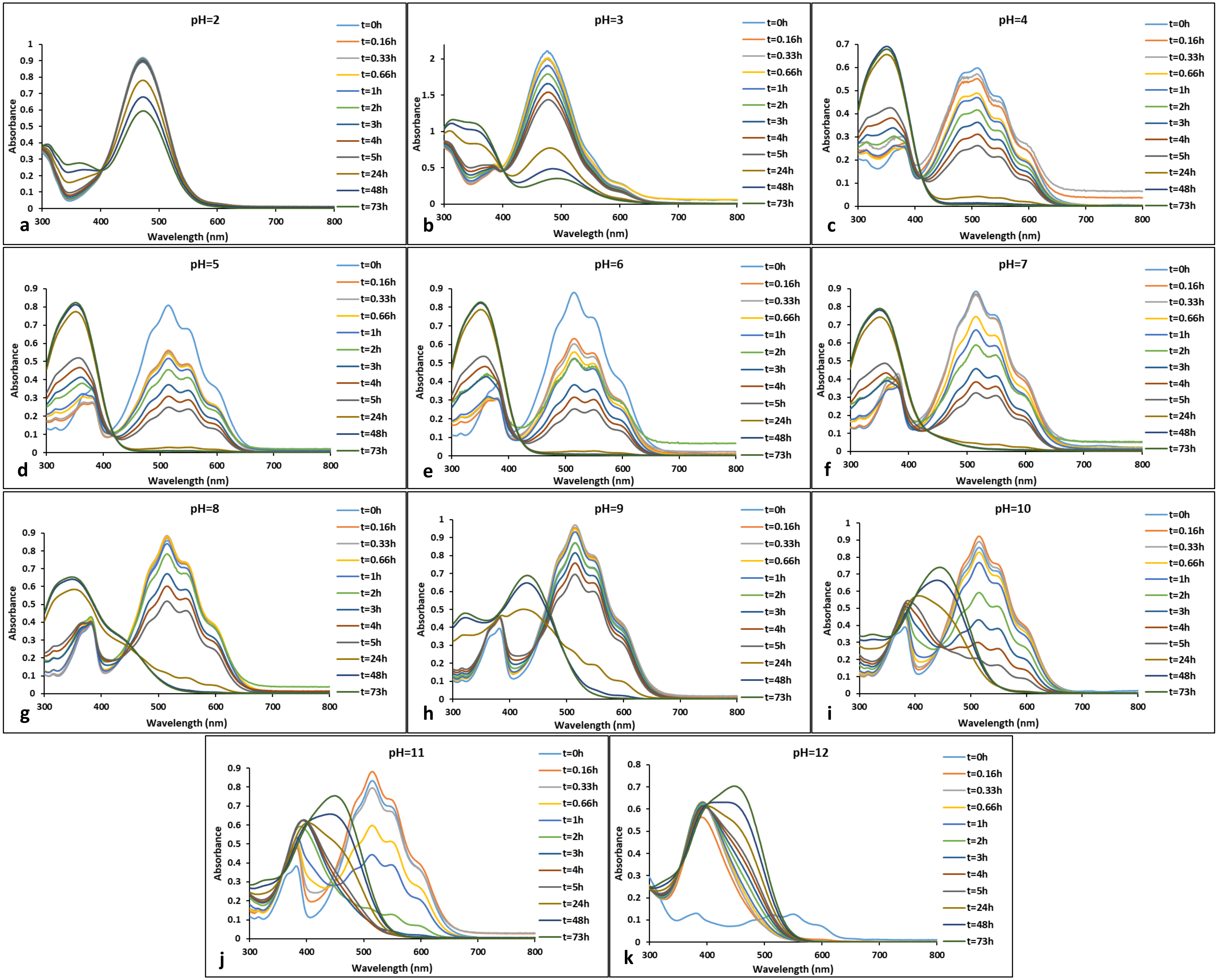


Figure S34. UV-Vis spectra of **5**

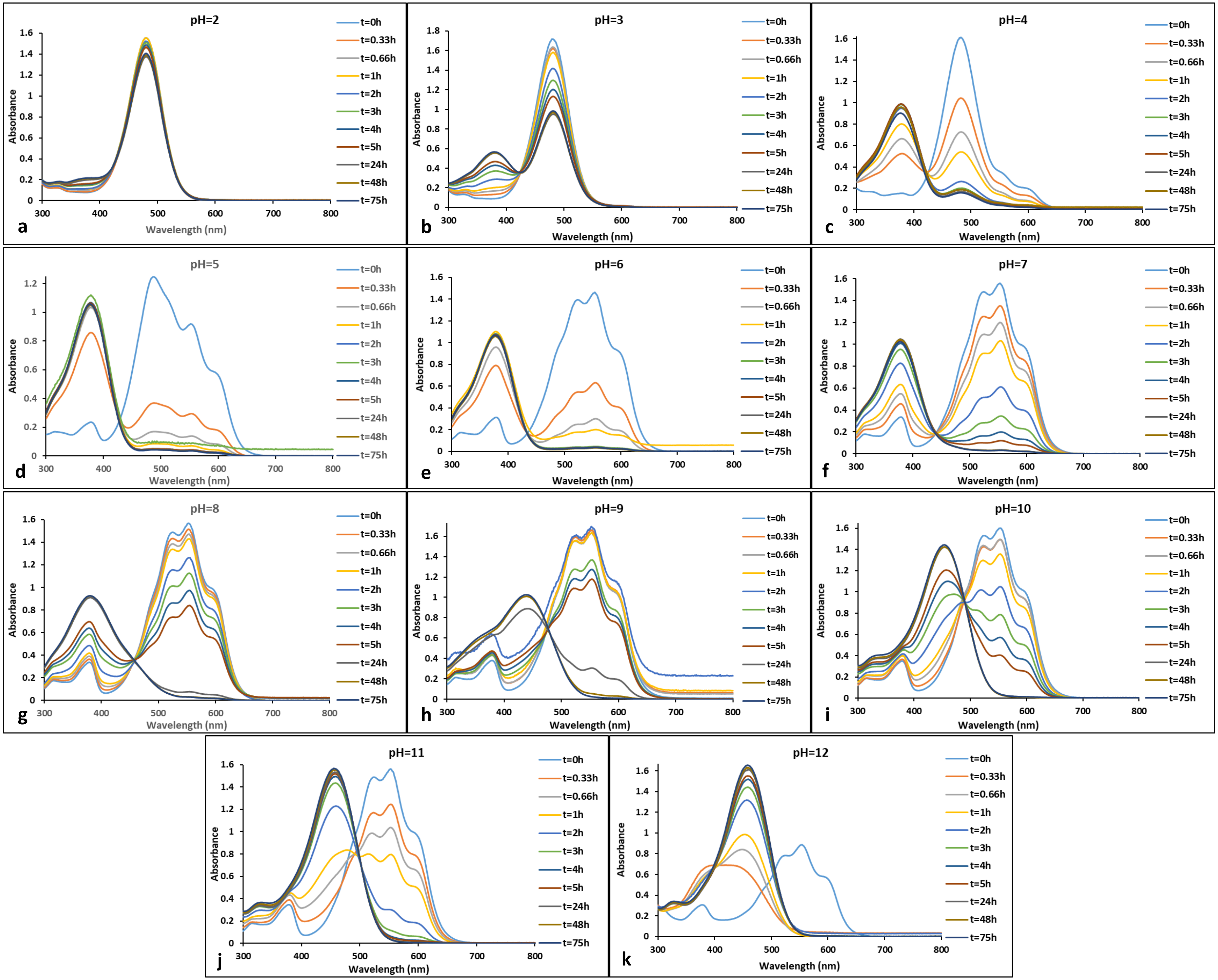


Figure S35. UV-Vis spectra of **6**
